# Integrated genetic and metabolic landscapes predict vulnerabilities of temozolomide resistant glioblastoma cells

**DOI:** 10.1038/s41540-020-00161-7

**Published:** 2021-01-08

**Authors:** Selva Rupa Christinal Immanuel, Avinash D. Ghanate, Dharmeshkumar S. Parmar, Ritu Yadav, Riya Uthup, Venkateswarlu Panchagnula, Anu Raghunathan

**Affiliations:** 1 Chemical Engineering Division, CSIR-National Chemical Laboratory, Pune, 411008 India; 2 Academy of Scientific and Innovative Research (AcSIR), CSIR-National Chemical Laboratory, Pune, 411008 India; 3 Present Address: Institute for Systems Biology, 401 Terry Ave N, Seattle, WA 98109-5263 USA; 4 Present Address: The Francis Crick Institute, 1 Midland Road, London, NW1 1AT UK

**Keywords:** Biochemical networks, Systems analysis

## Abstract

Metabolic reprogramming and its molecular underpinnings are critical to unravel the duality of cancer cell function and chemo-resistance. Here, we use a constraints-based integrated approach to delineate the interplay between metabolism and epigenetics, hardwired in the genome, to shape temozolomide (TMZ) resistance. Differential metabolism was identified in response to TMZ at varying concentrations in both the resistant neurospheroidal (NSP) and the susceptible (U87MG) glioblastoma cell-lines. The genetic basis of this metabolic adaptation was characterized by whole exome sequencing that identified mutations in signaling pathway regulators of growth and energy metabolism. Remarkably, our integrated approach identified rewiring in glycolysis, TCA cycle, malate aspartate shunt, and oxidative phosphorylation pathways. The differential killing of TMZ resistant NSP by Rotenone at low concentrations with an IC_50_ value of 5 nM, three orders of magnitude lower than for U87MG that exhibited an IC_50_ value of 1.8 mM was thus identified using our integrated systems-based approach.

## Introduction

The grand challenge of designing new therapies is the cellular complexity of a multi-hit, multifactorial disease like cancer and the rapid emergence of chemo-resistance^1–5^. Genome scale measurements allow cataloging drug and therapeutic candidates^6,7^. However, their integration with constraints-based metabolic modeling presents a paradigm shift in understanding homeostasis, disease, and treatment strategies^6,8^. Cancer cells are punctuated by their characteristic ability to proliferate and for unchecked proliferation in part due to mutation and in part due to disruption of homeostatic control coordinated by metabolic networks^9–14^ and unchecked signaling pathways^15,16^. In this work, we showcase unique therapeutic windows that exploit metabolic pathway vulnerabilities during cell growth to tackle chemotherapeutic resistance.

Glioblastoma Multiforme (GBM), is an aggressive brain cancer with inherent heterogeneity^17–19^. Temozolomide (TMZ), an alkylating agent is the most effective chemotherapeutic agent against GBM. However, TMZ resistance is increasing creating an urgent need to identify new therapeutic strategies^20–23^. Our previous study^24^ differentially analyzed growth limiting metabolites and nutrient preferences for respiration in TMZ resistant neurospheroidal cells (NSP) isolated from an authenticated cell-line population of U87MG cells. In this study, we have identified the Complex I inhibitor, rotenone as an alternate drug to induce killing of the drug resistant NSP. Our work highlights the use of constraints-based modeling integrated with exome, limited transcriptome and metabolome data to identify vulnerabilities of a chemo-resistant glioblastoma cell to identify differential response of an Electron Transfer Chain (ETC) inhibitor rotenone. We pioneer a scalable systems biology workflow from isolation of resistant heterogenous populations to molecular profiling/measurements integrated with metabolic modeling to rationally identify reprogramed pathways resulting in identifying potential alternate drugs.

## Results

### Differential metabolomic signatures of temozolomide sensitive and resistant cells

**Fig. 1:**
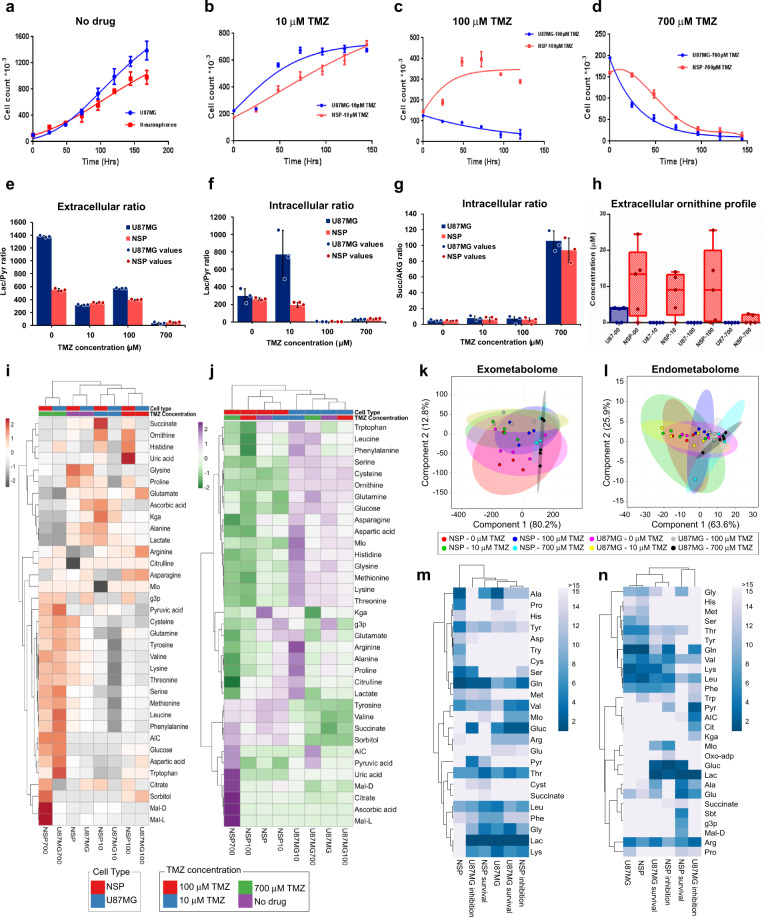
Metabolomic characterization of temozolomide resistance. Growth of U87MG and NSP in the absence of TMZ (**a**), in the presence of 10 μM TMZ (**b**), 100 μM TMZ (**c**), and 700 μM TMZ (**d**). The growth rates of U87MG and NSP were calculated using the Gompertz function (lines inside the dot plots) using GraphPad Prism Software. **e** Extracellular lactate (Lac) to Pyruvate (Pyr) ratio and **f** Intracellular Lac/Pyr ratio. Absolute concentration ratios (shown as bar plots) were calculated and plotted to delineate Warburg effect. **g** Intracellular succinate (Succ) to a-keto glutarate (AKG) ratio. The absolute levels were normalized to cell number to estimate the values plotted. **h** Extracellular ornithine levels. NSP cells show higher levels of Ornithine irrespective of TMZ treatment, a phenotype that is inherent in NSP. The boxes in the boxplot indicate the upper and lower quartiles of the data and the middle line is the median with the whiskers extending to 1.5× interquartile range. Dots are the sample data points (*n* = 5 for time points 0 h, 24 h, 48 h, 72 h, and 96 h). **i** Exo-metabolome profile and **j** Endo-metabolome profile comparison across U87MG and NSP cells. The heatmap is plotted using absolute concentration (C) differences between 96 h (t96) and 0 h (t0), and calculated as C_t96_–C_t0_. These values are processed to indicate *Z*-score (color scale) using Euclidean clustering. **k** Exo-metabolome PCA and (**l**) endo-metabolome PCA showing the clusters according to the TMZ concentrations used for treatment as 0 μM, 10 μM, 100 μM, and 700 μM. **m** and **n**. VIP scores for growth essential metabolites according to Exo-metabolome (**m**) and Endo-metabolome (**n**) profiles. Error bars indicate mean ± s.d.

We first analyzed the growth of U87MG and NSP glioblastoma cells in the presence of TMZ in varying concentrations (Fig. 1a–d). Growth was unaffected in both NSP and U87MG at 10 μM TMZ (Fig. 1b). U87MG had lowered growth rates in the presence of 100 μM TMZ (Fig. 1b; 2.5-fold change or 43% reduction) and showed a death profile at 750 μM TMZ (Fig. 1d). However, NSP continued to survive but showed consistently lower growth rates in the presence of 100 μM (Fig. 1c; 1.8-fold lower) and 750 μM TMZ (Fig. 1d; 2.3-fold lower). Differential consumption and release (CORE) profiles of metabolites that represents the exo-metabolite status after 96 h of growth (Fig. 1-i) is attributable to the drug dose response of TMZ that were identified from both the cell types. Lactate secretion was always higher in U87MG (Fig. 1e, f) when cells survived inspite of the presence of TMZ indicating a continued Warburg effect^12–14^ and efforts towards anabolic macromolecule biosynthesis. The drug dose dependent 3-fold higher lactate-to-pyruvate ratios in U87MG also indicated differential cytoplasmic NADH/ NAD^+^ ratios and altered redox state (Fig. 1e, f). This indicated an increase in the reduced forms of co-factor NADH (Supplementary Table 4) and a potentially compromised pyruvate dehydrogenase (PDH) activity. The increased NADH in U87MG during cell death suggests inability to recycle NAD^+^.

Increased intracellular succinate to AKG (SUCC/AKG) ratios were observed with increasing TMZ concentration and thus was a potential index of higher methylation and drug action resulting in lowered cell growth (Fig. 1g, Supplementary Figs 4, 5, and 6). Time dependent growth profiling indicates maximal differential cell counts between 24 and 48 h. This corresponds to a maximum fold change (1.4 to 1.7-fold) in SUCC/AKG ratios. With decrease in growth profile differences, SUCC/AKG ratios also reduce (Supplementary Fig. 6). Succinate levels were increasingly higher in U87MG and lower in NSP with varying TMZ concentrations (Supplementary Fig. 5). Increased AKG (Supplementary Fig. 4) in NSP could be a result of preferred glutamine metabolism^24^. Extracellular Ornithine levels (Fig. 1h, Supplementary Fig. 1) were drug independent and consistently lower in U87MG with a higher consumption rate and depleted completely in presence of TMZ in contrast to NSP (Fig. 1h). Higher ornithine levels in NSP could potentially be through an operational urea cycle and related pyrimidine nucleotide metabolism. Principal Component Analysis (PCA) for CORE profiles were shaped by increasing TMZ concentrations for both U87MG and NSP cells (Fig. 1k). Figure 1l summarizes through a PCA plot differential endo-metabolite levels from initial time points indicating varying intracellular *milieu* that dictates the differential metabolism and response to drug. The heatmap of the exo-metabolite profiles was plotted using absolute concentrations (Fig. 1i) to identify maximum impact at 700 μM TMZ on the CORE of metabolites by U87MG and NSP. A drug induced increase in pyruvate concentrations in U87MG alone, indicates potentially higher NAD^+^ recycling. Varied extracellular microenvironments (Supplementary Fig. 1) were observed as succinate, ornithine, histidine and uric acid have increased secretion in NSP while arginine, asparagine and glutamate are higher in U87MG. Differential dynamics of metabolites showed varied glutamine, glutamate and AKG phenotypes across U87MG and NSP (Supplementary Fig. 1). Intracellular profiles and clustering data indicate metabolic reprogramming between U87MG and NSP independent of drug dose (Fig. 1j, Supplementary Fig. 2).

**Fig. 2:**
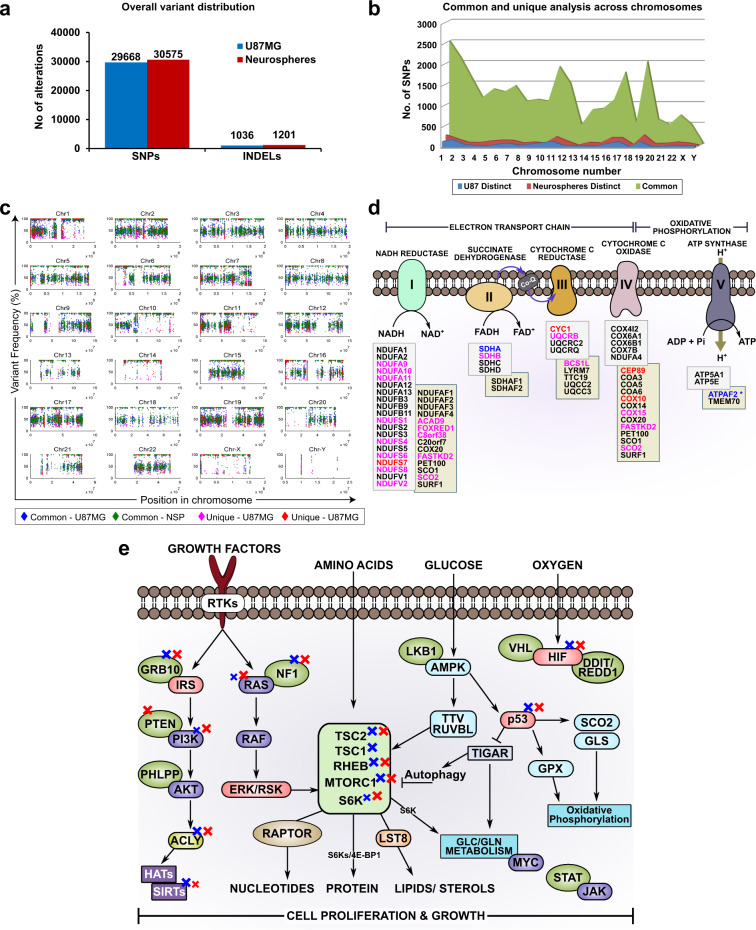
Exome characterization of U87MG and NSP. **a** Variant distributions in U87MG and NSP. **b** SNPs that are common and unique across the TMZ sensitive (U87MG) and resistant (NSP) cells. **c** Chromosome-wide distribution of mutations. **d** Electron transport chain complexes and their associated mutations profiled using exome sequencing—blue fonts indicate mutations in U87MG, red fonts indicate mutations in NSP and pink fonts indicate mutations in both cell types. **e** Signaling network perturbations that play a key role in controlling metabolism. A blue cross indicates a mutation in U87MG, A red cross indicates mutation in NSP, size of the cross is scaled based on the number of mutations (either equal sizes or different).

Concentrations of amino acids including glutamine, serine, tryptophan were higher intracellularly in U87MG while succinate, malate, citrate/isocitrate and ascorbate were higher in NSP indicating a higher TCA cycle flux (Supplementary Fig. 2). Partial least squares discriminant analysis (PLS-DA) and VIP score plots (Fig. 1k–n) shows glutamine as critical to growth/survival of NSP in the extracellular microenvironment (Fig. 1m) and lactate and glucose critical to that of U87MG. Glucose, glutamate and lactate become critical intracellularly (Fig. 1n) for NSP survival in the presence of TMZ (Supplementary Fig. 2). The circulation of lactate into alanine via the Cori cycle potentially becomes important for NSP for survival in the presence of TMZ. Aromatic amino acids seem to be more critical for growth of NSP than U87MG. Malate is potentially critical to survival of NSP suggesting a functional Malate-aspartate/pyruvate shunt, also validated by exome data (Fig. 2d) and in silico predictions (Fig. 4). Dynamic metabolite profiles and CORE for glutamine, serine and tryptophan in the presence of temozolomide compared to glucose further indicate a potential switch in substrate uptake and metabolism in NSP (Supplementary Fig. 1). The simultaneous measurement of respiration and growth profiles through Biolog^TM^ phenotype microarray testing also identified differential coupling between growth and respiration on 57% of the C/N sources tested including glutamine in NSP^24^ (Supplementary Figs 11–14).

### Genotypic landscape dictates heterogeneous states of temozolomide response

**Fig. 3:**
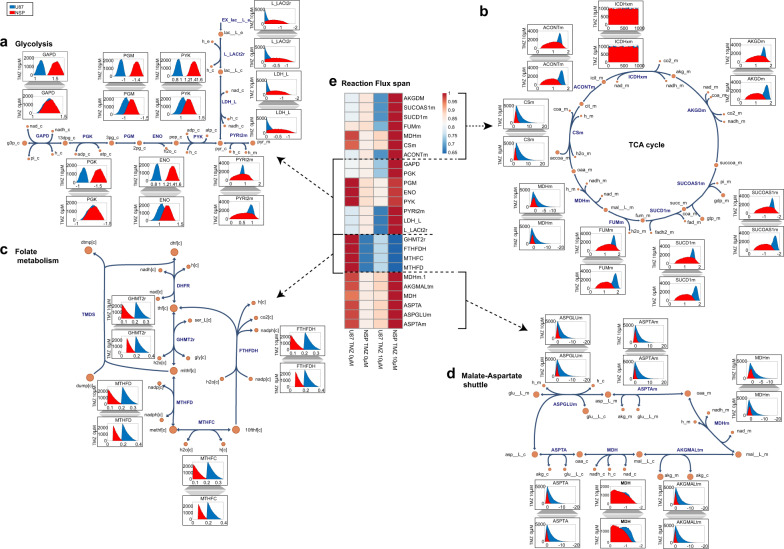
Constraints-based metabolic modeling of Glioblastoma predicts targetable alternate flux distributions in the metabolic pathways of temozolomide resistant cells. **a-d** Differential predicted flux spans and probability of flux distribution using monte-carlo sampling (blue–U87MG; red–NSP) for (**a**) Glycolytic pathway. **(b)** TCA cycle. **(c**) Folate metabolism and (**d**) Malate-aspartate shuttle. **e** Differential flux spans predicted in silico using flux variability analysis (FVA) for U87MG and NSP associated with reprogrammed pathways (arrows point towards their associated pathways showing predicted probability of flux distribution).

In order to see how genotype was driving metabolic reprogramming associated with TMZ response and growth, we sequenced the exome of U87MG and NSP cells (Fig. 2, Supplementary File 3). Curating the distribution of mutations (Supplementary Fig. 8) identified 29668 and 1036 Single Nucleotide Polymorphisms and indels in U87MG and 30575 SNPs and 1201 indels in NSP (Fig. 3a). Novel mutations not reported in dbSNP and COSMIC databases were identified as 1804 and 795 in U87MG and NSP, respectively, with greater than 97% reported in both the databases (Supplementary Fig. 7). Of the total 12,130 genes that harbored sequence changes, 397 genes and 1893 associated SNP mutations were unique in U87MG while 559 genes and 2804 associated SNPs were unique in NSP (Supplementary Fig. 8). All the genetic variations were distributed across each chromosome without bias; Chromosomes 2, 3, 17, and 19 harbored maximum sequence alterations (Fig. 2b, c). Functional annotation (Oncotator derived) of genomic alterations identified maximum (>9000) missense mutations changing protein sequence and driving functional change. About 8% of the metabolic genes mutated were unique to NSP while only 3% were unique to U87MG (Supplementary Fig. 9). PolyPhen (PPH2; Polymorphism Phenotyping)^25,26^ was used for annotating and predicting potential impacts of mutations on protein structure/function of all identified SNPs in coding genes for U87MG and NSP (Supplementary File 3).

Signaling genes associated with substrate uptake, central metabolism, electron transport, respiration and growth were mutated in U87MG and NSP (Fig. 3d, e). The functional impact of these mutations was calculated using PPH2 to have a deleterious effect in most cases (Supplementary Table 1). Amino acid transporters SLC38A3, SLC38A4, SLC1A5 varied in NSP alone, explaining differential CORE profiles. Such changes could result in functional changes including differential transport mechanisms or metabolite pathway utilization as shown in the differential growth and respiration profiles on selected nutrients (Supplementary Fig. 11 and S15). ABC transporters involved in drug efflux are differentially expressed and impact drug transport and growth rates in NSP and U87MG^24^ (Supplementary Fig. 19; Supplementary File 4). Unique mutations were identified in negative regulators PTEN and TSC1 for NSP and U87MG, respectively, in receptor tyrosine kinase PI3K-AKT-mTOR pathways. mTOR, a gene that is linked to cell cycle progression by regulating cell growth, has a significant role in controlling metabolic homeostasis at the organismal level^27^. mTORC1 signaling can be affected by mutational, transcriptional or translational changes in genes that result in activation or loss of function of both positive and/or negative regulators. These include but are not limited to receptors of growth factors, tyrosine kinases, PI3-kinase, Akt, mTOR, PTEN, LKB1, RHEB, TSC1, TSC2, S6K (Fig. 2e; Supplementary Fig. 9). The SIRTUIN genes (SIRT4/6) responsible for NAD/NADH sensing were differentially mutated and control differential glutamine metabolism (Figs. S11 and S15).

Electron transport chain/Oxidative phosphorylation genes were extensively mutated with unique mutations in U87MG only in Complex II (sdhA) and Complex III (CYC1). ATPase (ATP4A) harbored unique mutations (Fig. 2d, Supplementary File 3) in NSP alone. The higher levels of intracellular succinate in U87MG are explained by the unique mis-sense mutation (dbSNP #rs76896145) in mitochondrial complex II (succinate dehydrogenase, sdhA). This validates the PPH2 likelihood prediction (probability of 0.998 and FDR of 0.044; (Supplemental Supplementary Table 1). Functional impact of the p.S456L mutation on sdhA activity is evident in the differential respiration and growth levels of U87MG AND NSP on succinate as sole carbon source in the BIOLOG^TM^ phenotypic microarray data (Supplementary Fig. 7A). The unique mutation in U87MG is a potentially compensatory mutation that allows growth on succinate in the BIOLOG^TM^ profiles (Supplementary Fig. 7B). The oxidation of succinate to fumarate (tricarboxylic acid (TCA) cycle) is catalyzed by sdhA, carrying the unique mutation in U87MG. A mutation can disrupt electron flow through the iron-sulfur clusters of sdhB, which is anchored to the inner mitochondrial membrane by sdhC and sdhD subunits, ultimately disrupting the flow to the ubiquinone pool to generate ATP. AKG-dependent histone demethylases involved in epigenetic regulation of oncogenes and tumor suppressor genes are potential targets for inhibition by succinate accumulation.

There is a mutation in Cytochrome C-1 (Cyc1 gene) one of the 11 genes in the mitochondrial respiratory chain (MRC) complex III (Supplementary File 3). The unique mutation in the Cyc1 gene in NSP has an impact on respiration as seen in the cellular reductase profile obtained using microarray phenotyping for D-actinomycin (Supplementary Fig. 19). Due to its localization on the trans-inner-membrane, a mutation can disrupt the Q-cycle mechanism and coupling associated with electron transfer from ubiquinol (reduced coenzyme Q or CoQ) to cytochrome c accompanied by proton translocation from the mitochondrial matrix to the intermembrane space. Complex III is critically placed at the crossroads of glycerol-3-phosphate dehydrogenase, dihydroorotate dehydrogenase (DHODH), electron transfer flavoprotein (ETF) and sulfide–quinone reductase (SQR), all alternate electron transfer pathways that lead to CoQ^28^. Such a disruption could result in an increase in AMP to ATP ratio, thus activating AMPK. Mutations in Adenylate kinase ADK could modify the ratios of ATP, AMP, and ADP resulting in impact of p53 and AMPK signaling. A differential upregulation or expression of genes from these pathways^24^ is an indication of the known crosstalk between AMPK and the cellular survival axis of PI3K/Akt/mTOR pathway

OAZ1 the controller of the enzyme Ornithine Decarboxylase (ODC1) regulating polyamine synthesis and associated cell growth was mutated in U87MG alone (Supplementary File 3) supporting differentially lower levels of ornithine. The frameshift in the ODC protein at p.F219fs Chr 2, position #10583624 potentially impacts dimer formation decreasing ornithine levels as observed in U87MG compared to NSP cells (Supplementary Fig. 10).

ACO1, an isozyme for aconitase, harbors a missense mutation (Supplementary Table 1, Supplementary File 3) supporting the increased citrate/isocitrate accumulation in NSP that potentially shaped higher IDH1 gene expression^24^. Unique mutations were also identified in NSP in Glutathione metabolism (GCLM, GGT2, GPX1) (Supplementary File 3). These mutations may uniquely modify the processes of GSH synthesis (ATP dependent) and degradation required to be in strict balance and control for normal intracellular processes. The activity of Glutathione peroxidase-1 (GPX1) can prevent DNA damage and inhibit synthesis of inflammatory mediators such as prostaglandins and leukotrienes. There is a possible relationship between the activity of GPX1 and concentrations of selenium binding protein (SBP1). SBP1 potentially reduces the activity of GPX1. GPX1 is a selenoprotein that catalyzes the glutathione dependent reduction of organic hydroperoxides and hydrogen peroxide (H2O2). Sodium selenite is known to increase GPX-1 protein and activity in a dose-dependent manner^29^ and attenuating oxidation of NADPH and NADH^30^. Low concentrations of Sodium selenite have a higher impact on respiration in U87MG than NSP (Supplementary Fig. 17). An increase in the sodium selenite concentration impacts NSP more drastically as compared to U87MG, lowering the NAD production. The unique mutation in Guanylate cyclase (GC) beta subunit in NSP, indicates potential change in NO metabolism modulating downstream signaling. Differential NO levels could impact mitochondrial respiration differentially through unique mutations in Complex IV, COX4 (Fig. 2d). This could potentially reduce ATP synthesis^31^ and impact respiration coupled growth^24^ (Supplementary Figs 11–14).

### Constraints-based metabolic model predicts alternate flux distributions in central metabolic pathways in temozolomide resistant cells

**Fig. 4:**
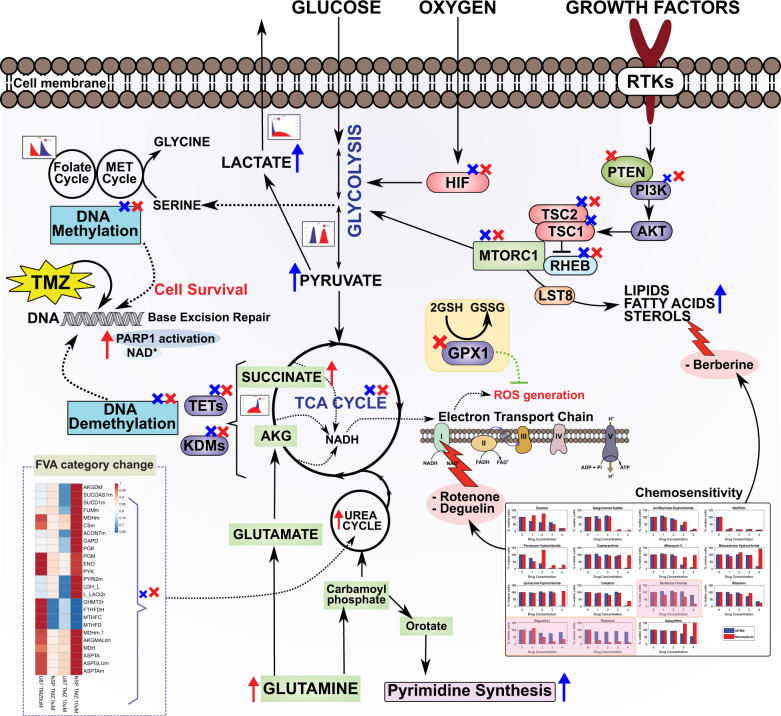
Potential metabolic reprogramming in temozolomide resistant cells. Metabolic rewiring via candidate genes, metabolite levels, and their effect on controlling major metabolic pathways in TMZ resistance are highlighted by differential mutations, differential reaction flux from in silico predictions ((blue–U87MG; red–NSP) resulting in differential drug molecule chemo sensitivity. The most impactful drugs are those targeting reprogrammed pathways. A blue cross indicates a mutation in U87MG, A red cross indicates mutation in NSP, size of the cross is scaled based on the number of mutations (either equal sizes or different).

A central metabolic reconstruction of human metabolism^32^, containing 380 reactions, was contextualized to represent sensitive and resistant glioblastoma cells in silico using experimentally determined metabolite uptake rates (Supplementary File 5) from dynamic concentration profiles, growth rates and enzymopathies from exome sequencing. A cell specific biomass composition was determined for U87MG and NSP taking into account literature mass fractions of different macromolecules such as lipid, protein, DNA, and RNA and cell dry weights^32^ (see “Methods” section). The model was validated by estimating uptake rates of primary nutrients with more than 90% accuracy (Supplementary Table 3). Lower oxygen uptake rates were predicted to support experimental growth and substrate uptake rates for NSP as compared to U87MG indicating potentially a more hypoxic micro-environment for chemo resistant NSP than U87MG. Using an in silico NADH oxidase to constrain a core model of glioblastoma metabolism predicted more NAD+ recycling for U87MG (NADH oxidase flux higher) than NSP (Supplementary Fig. 20A, Supplementary File 5). This was validated by differential NADH concentrations and NAD/NADH ratios observed (Supplementary Table 4). Flux variability analysis identified differential flux span/ranges (Fig. 3e) for U87MG and NSP in reactions related to TCA cycle and glycolysis subsystems and the folate and the malate-aspartate shuttles. The bidirectional ornithine carbamoyl transferase reaction in U87MG is forced unidirectionally in NSP explaining the potential downstream effects of ODC1 gene mutation and ornithine levels. Reprogrammed metabolic network states assessed through Monte Carlo sampling (see “Methods” section) of the solution space of flux distributions identified differentially correlated sets, one forming a micro cycle leading to proline/ornithine and cholesterol metabolism in NSP alone (Supplemental File S5). The upstream precursors (acetate, acetoacetate, hydroxybutyrate) (Supplementary Fig. 16) and downstream metabolite vitamin D (Supplementary Fig. 18) of the cholesterol pathway have differential impact on growth of U87MG and NSP. There was a differential probability of flux distribution and the magnitude of flux in the contextualized cell specific in silico U87MG and NSP models. Pathways including glycolysis, TCA cycle, folate, and malate/aspartate shuttle (Fig. 3a–d) in U87MG and NSP were reprogrammed. A drug dependent differential probability of flux distribution within the cell lines was also evident (Fig. 3e). Folate pathways including serine and glycine metabolism also have higher probability of higher flux in U87MG indicative of increased probability of methylation and TMZ response. The in silico predictions thus indicate reprogramming of metabolism (Fig. 4) validated through rigorous experimentation between glioblastoma cells that are sensitive and resistant to the drug. Thus, drugs that can impact these reprogrammed pathways including cholesterol lowering and oxidative phosphorylation limiting agents, are potential targets for TMZ resistant NSP.

## Discussion

Cells of neuronal origin rely on oxidative phosphorylation to meet energy demands. Glycolysis and TCA cycle are major pathways providing metabolic precursors for biosynthesis and energy production. The most significant pathways involved in metabolic adaptation in cancer cells seem to be aerobic glycolysis, glutaminolysis, and mitochondrial oxidative phosphorylation. All these pathways have higher flux distributions and activity in TMZ sensitive U87MG as compared to TMZ resistant NSP, as identified through our integrated analyses (Fig. 4). The activities and metabolic flux of these pathways are critically tuned to ensure optimal nutrient allocation and distribution for cellular proliferation, growth and function. This reprogrammed metabolism observed in U87MG, also seen in other cancers^9^, potentially undergoes another round of reprogramming in NSP to become resistant to the drug, TMZ. These include pathways related to carbon metabolism (nutrient uptake, growth signaling, glycolysis, TCA cycle, 1C methionine, and folate cycles), Nitrogen metabolism (Urea cycle, pyrimidine synthesis), and energy metabolism (oxidative phosphorylation and ETC) (Fig. 4). The integrative analysis of these pathways showcases the tacit connections and cross talk between metabolism, methylation, growth and drug response.

Constraints-based modeling elucidated the novel mechanisms of TMZ resistance and suggested that the drugs impacting cholesterol lowering and oxidative phosphorylation would arrest the NSP survival. In a follow up screen in a 92 cytotoxic drug panel of phenotypic microarray analysis (Supplementary Fig. 19), Berberine, Deguelin, and Rotenone were identified as effective against NSP alone (Fig. 4 inset) validating the predicted modeling results. Detailed drug response profiles of rotenone were established for both NSP and U87MG. Significantly, the IC_50_ values were identified as lower by an order of magnitude calculates as 5 nM for NSP as compared to 1.8 mM for U87MG (Supplementary Fig. 20B, C). The synergistic use of rotenone also reduced the dose of TMZ required for cell death as identified by metabolic reprogramming through integrated constraints-based analysis (Supplementary Fig. 21). A combination of temozolomide and rotenone exhibited a positive synergy on both U87MG and NSP. At 5 nm Rotenone, the sensitivity to temozolomide was restored and the IC_50_ for TMZ was 1000-fold lower for NSP (Supplementary Fig. 21B). Higher concentrations of Rotenone reduced the IC_50_ dose value of TMZ in U87MG to 7-fold (Supplementary Fig. 21A).

Taken together, our approach of integrated constraints-based analysis identified the critical role and vulnerability of Complex I of the ETC in survival of NSP and thus helped identify rotenone as an alternate drug for inducing death of TMZ-resistant NSP. The adaptation or evolution of NSP, the resistant cell, in the presence of distinct selection pressures (therapeutic drug, TMZ), towards a behavior and metabolism closer to the normal cell function indicated by increased oxidative phosphorylation and the balance between glycolysis and mitochondrial oxidative phosphorylation is essential. Such integrated systems level approaches, are essential to unraveling the tacit connections between epigenetics, metabolism and genotyping and are scalable to the clinic to fill a critical need for predictive models in individualized therapy. Regardless of the requirement of rigorous characterization in animal models and clinically derived cell lines to extend the promise of this study, we foresee the capability of these approaches to expedite choices for personalized medicine.

## Methods

### Cell culture

U87MG cell line (HTB-14; Human Glioblastoma Multiforme from ATCC) was cultured in DMEM (Dulbecco’s Modified Eagle’s Medium, Gibco) with Glucose (1 mg per mL) and L-glutamine (0.584 mg per mL). 10% fetal bovine serum (FBS, Gibco^TM^, ThermoFisher Scientific) and 1% non-essential amino acids (Sigma-Aldrich) was used additionally for growth. Cell lines were maintained at 37 °C in a humidified atmosphere of 5% CO_2_/95% air. For the separation of NSP, the sub-population sorting assay was performed for Fluorescence-activated cell sorting (FACS) with cells at 70–80% confluency using BD FACSAria III (BD biosciences Pvt. Ltd) and separated the populations using Hoechst 33342 staining procedure^24^. The detailed protocol is available in our previous study^24^. After separation using FACS, NSPs were initially maintained in neurobasal medium (Gibco^TM^, ThermoFisher Scientific) supplemented with B27 supplement (Gibco^TM^, ThermoFisher Scientific), 0.2 μg per mL of epidermal growth factor, EGF (ThermoFisher Scientific) and 0.2 μg per mL of basic fibroblast growth factor, bFGF (ThermoFisher Scientific). Further sub-culturing and passaging of NSP was carried out using a similar medium as U87MG to avoid any contribution from different micro-environments in delineating heterogeneity of molecular signatures. NSP were cultured as free-floating spheres in appropriate low attachment T-75 flasks or 6 well or 24 well plates (Nunc^TM^, ThermoScientific^TM^) for this study.

### Growth and temozolomide dose response curves

Growth of the cells (U87MG and NSP) were studied by monitoring their proliferation via cell count over a period of 168 h (7 days). The initial seeding set had a starting population (*N*_*o*_) at ~10,000 cells per well. The growth profile was studied in a 24 well plate (Nunc tissue culture-treated) for ease of harvesting. Both U87MG and NSP cells were harvested every 24 h and counted using a hemocytometer based on the trypan blue dye exclusion assay. Before counting, the NSP population was also disaggregated by trypsinization. For dose-response experiments, cells were plated in replicates at ~20,000 cells per well in 24-well plates (Nunc^TM^ tissue culture treated, ThermoScientific^TM^) in full growth medium for 24 h and then treated them with different doses of TMZ (10 µM, 100 µM, and 700 µM). Three biological replicates were performed with three technical replicates in each biological replicate on a 24-well plate (Nunc^TM^ tissue culture treated, ThermoScientific^TM^). Growth and temozolomide response curves were graphed with the number of cells on the *Y* axis and time on the *X* axis. The data was fitted using a logistic Gompertz function using GraphPad Prism software and the growth parameters were calculated.

### Metabolite profiling using liquid chromatography-high resolution mass spectrometry (LC-HRMS):

*Sample extraction, dilution, and internal standard spiking*. Eight samples (collected at the end of every 24 h of growth curves) from each experiment setup (Without drug, 10 µM TMZ, 100 µM TMZ, and 700 µM TMZ) were harvested over a period of five days and used for the metabolic profiling to understand nutrient uptake and release kinetics. A sample pooling strategy^33^ was applied to reduce the number of samples and for high-throughput quantification. A viable cell count was performed to count the number of cells in each sample from growth curves. After cell count, the samples were centrifuged at 2795 × *g* (4 °C). The supernatant was used for extracellular analysis and the pellet was used for intracellular analysis.

*Extracellular samples.* Each replicate sample was prepared and stored at −80 °C; thawed on an ice bath to aliquot 100 µL of sample for extraction. The aliquot was transferred into a fresh 1.5 mL centrifuge tube. Four hundred microliter of chilled methanol (previously stored in −80 °C) was added. The solution was thoroughly mixed for 2 min followed by centrifugation for 15 min at 2795 × *g* (4 °C). The tubes were carefully removed, 300 µL of supernatant was withdrawn and transferred into a fresh tube (Dilution level: 5×).

*Dilutions.* A two-step serial dilution of supernatant was performed using 50% acetonitrile in water. In the first step, 50 µL of sample was thoroughly mixed with 450 µL of diluent (Dilution level: 50×). This solution was further diluted by mixing 100 µL of sample solution with 400 µL of diluent (Dilution level: 250×). Before injection, 100 µL of sample solution was mixed with equal volume internal standard solution containing 4.4 µM verapamil in 50% acetonitrile in water with 0.2% formic acid.

*Intracellular samples*. The cell pellet was washed with PBS (Phosphate buffer saline) (pH–7.4) by centrifugation at 2795 × *g* (4 °C). This step was repeated twice to remove any media residues. In the subsequent steps, HPLC grade methanol (100% methanol on dry ice for the first round, and 80:20 methanol/water at 4 °C for the next two rounds was used for extracting metabolites. The extracted samples were stored in aliquots at −80 °C until use. All data collected from LCMS analyses are provided in Supplementary File 2.

*LCMS parameters setup and software details*. The UPLC and MS was operated using Xcalibur (Thermo Fisher Scientific Pvt. Ltd., Version 2.0) software platform, whereas HESI source parameters were set using Tune module (Thermo Fisher Scientific Pvt. Ltd., version 2.1). Samples were stored in temperature controlled Accela autosampler maintained at 4 °C during LC-HRMS analysis. A reverse-phase C18 hypersil gold column (10 cm × 2.1 mm × 3.0 μm) was used for chromatography. The mobile phase consisted of 0.1% formic acid in deionized water (Mobile phase ‘A’) and 0.1% formic acid in acetonitrile (Mobile phase ‘B’). The elution gradient was set as 70% of mobile phase A (0.0–2.5 min), 10% A (3.5–5.5 min), 70% A (5.5–8.0 min) with a constant flow rate at 1000 μL per min. Five microliter of samples was injected for analysis using the auto-sampler unit. The data was acquired in both positive and negative ion mode in two separate batches. Metabolomics data analysis was carried out by the Qual and Quan browser modules of Xcalibur (Thermo Fisher Scientific Pvt. Ltd.). The same protocol was followed in our previous study^24^.

### CORE (consumption and release) clustering and PCA (principal component analysis)

The concentrations for all 35 metabolites identified from LC-MS/MS analysis were further clustered using ClustVis web tool (http://biit.cs.ut.ee/clustvis/). Consumption and release concentrations were calculated by subtracting the concentration at 0 and 96 h. These values were used as inputs for generating heat maps and for PCA. Heat maps and clustering was performed with the criteria of unit variance scaling (applied to rows) where all 35 metabolites were the rows in the heat maps. Both rows and columns were clustered using correlation distance and average linkage with the tightest cluster first in tree ordering. PCA was carried out by using SVD with the imputation algorithm specified in the ClustVis tool. In some specific cases (BIOLOG^TM^ experiments), the Euclidean algorithm was used for clustering to interpret the data. Variable Importance in Projection (VIP) scores and PLS-DA were calculated using MetaboAnalyst, a web-based metabolomic analysis tool^34–37^.

### Genomic DNA extraction and Exome sequencing

The complete/genomic DNA extraction from cultured U87MG and NSP cells was performed using DNeasy Blood and Tissue Kits Spin-column protocol (Qiagen, India). A total of 4 × 10^6^ each U87MG and NSP cells were centrifuged for 5 min at 300×*g* and resuspended in 200 μL PBS each. Twenty microliter proteinase K was used in the initial step to lyse the cells. The manufacturer’s protocol was followed. 4 μL RNase A (100 mg per mL) was used to remove any RNA contamination from the extracted DNA by incubating for 5 min at room temperature. ~15 µg of genomic DNA was extracted from each sample. This extracted DNA was used for the exome sequencing. Exome workflow of Ion Proton^TM^ systems (Life technologies Pvt. Ltd., India) was used to obtain the exome sequences of U87MG and NSP cells. Ion TargetSeq^TM^ Exome kit and Ion Proton^TM^ sequencer was used for acquiring the exome data that was further processed through TorrentSuite and Ion Reporter software to identify the variants and for the coverage analysis (Supplementary File 3).

### Functional annotation of Exome data

The variants of U87MG and NSP cells thus identified by exome sequencing had been analyzed for its functional effect using Oncotator web tool (https://portals.broadinstitute.org/oncotator/). Oncotator is a web-based application commonly used for annotating human genomic point mutations including Single Nucleotide Polymorphisms (SNPs) and Insertions and Deletions (INDELS). This includes genomic annotations (Gene, transcript, and functional consequence annotations for hg19 database), protein annotations (Site-specific protein annotations from UniProt, functional impact predictions from dbNSFP and cancer variant annotations), and common SNP annotations from dbSNP database. The input file contained the details of the position in chromosomes, reference and its corresponding variants identified that was uploaded in the web tool for analysis. The output file had the results with details of the gene name, gene IDs, HUGO symbol, variant classifications (Silent, 5’-UTR, 3’-UTR, Intron, Missense, Frame_Shift_deletions etc.), gene description, protein/amino acid change and its biological functions. These details helped further analysis and interpretation (Supplementary File 3).

### Phenotype microarray analysis

Biolog Phenotype MicroArrays™ (PM-M1 to PM-M14) from Biolog, Inc. USA (www.biolog.com) consist of panels of PMM screening assays—(i) Energy metabolism pathways; (ii) Ion and hormone effects on cells and (iii) Sensitivity to anti-cancer agents. In PM-M1 to PM-M4, the metabolic pathway activities were assayed by using the cell suspension (~20,000 cells/well) prepared in an inoculating fluid (IF-M1 or IF-M2) that lacks carbon and energy sources (provided with the BIOLOG^TM^ plates). Biolog Redox Dye Mix MA or Biolog Redox Dye Mix MB, was added to all wells. This measurement employs a tetrazolium dye that can be reduced to a purple formazan that can be measured at 590 nm with a microplate reader. The redox energy produced when a cell metabolizes a substrate is used to convert the color from yellow to purple formazan. The rate of formazan production changes linearly with time and corresponds to the number of viable cells. iMark™ Microplate Absorbance Reader (Bio-Rad), with a wavelength range of 400–750 nm, was used in our study to measure the absorbance. Cell suspension (~20,000 cells per well in a culture medium that is serum-free and containing D-glucose and L-glutamine was used for PM-M 5 to 8 and PM-M 11 to 14. All plates were incubated at 37 °C in a CO_2_ incubator. The absorbance readings were measured after an initial incubation (48 h) of cell growth, followed by adding the dye and reading for 24 h of study (with initial intervals at 15, 30, 45, and 60 min; 1 h intervals from 2 to 6 h; and final reading at 24 h of incubation).

### Drug dose response curves for Rotenone

For dose response experiments, four replicates at 20,000 cells per well were plated in 96 well Nunc^TM^ tissue culture plates for U87MG and low attachment ones for NSP in complete growth medium for 24 h, treated with different concentrations of rotenone followed by cell viability tests using MTT assay. Appropriate cell controls (without rotenone treatment) were used to estimate IC_50_ dose value. The concentrations of rotenone used in the IC_50_ estimation for U87MG cells were 0.5 mM, 0.1 mM, 1 mM, 2 mM, 3 mM, 4 mM, and 5 mM. The concentrations of rotenone used in the IC_50_ estimation for NSP cells were 5 nM, 10 nM, 20 nM, 50 nM, 100 nM, 0.5 mM, and 1 mM. The results were graphed and drug dose response parameters were calculated using Graph Pad Prism.

### Flux balance analysis using constraints based metabolic network analysis

A published model for central core metabolism^32^ consisting of 386 reactions (Supplementary File 5) that are highly conserved in cancer, was used to build U87MG and NSP specific models. The model consisted of reactions involved in metabolic functions such as, biomass precursor synthesis, core energy metabolism, co-factor transfer, regeneration reactions, and relevant pathways for high secretion/uptake metabolites etc. Cell specific biomass composition was determined for U87MG and NSP taking into account literature mass fractions of different macromolecules such as, lipid, protein, DNA, and RNA^32^. Specifically, protein dry weight fraction was assumed to be 70% of dry cell weight and DNA mass fraction was calculated considering 44 chromosome units for the U87MG as reported by ATCC records. While the remainder of biomass fraction was distributed amongst lipid and RNA as per literature reported values^32^.

Metabolic fluxes were estimated using flux balance analysis for these models by solving following linear Eq. 1.

1 $$Z_0 = {\mathrm{max}}\left\{ {c.v} \right\}$$

subject to,

2 $$S.v = 0$$

3 $$v_i^{{\mathrm{min}}} \le v_i \le v_i^{{\mathrm{max}}}$$

where, *S* is the stoichiometric matrix, *c* corresponds to the vector of objective applied to individual reaction, *v* is the reaction flux vector and *v*_*i*_^min^*, v*_*i*_^max^ denotes the bounds for *i*^th^ reaction flux range.

Systemic effects of genomic variants on cellular metabolism are well known. In order to incorporate enzymopathic effects of system specific unique mutations for these two cell lines, a list of reactions was identified (as shown in Supplemental file S5 following gene-protein-relation having genes with mutations having deleterious effects predicted by PPH2. This method uses the Naives Bayes approach coupled with entropy-based discretization that predicts a likelihood that the mutant allele impacts protein function, phenotype or fitness i.e., is the mutation benign or damaging. Such intracellular reaction’s flux bounds were constrained following Eq. 2^38^.

4 $${\mathrm{new}}V_{\left( {i,{\mathrm{max}}} \right)} = v_{\left( {i,{\mathrm{min}}} \right)} + 0.25 \times {\mathrm{abs}}\left( {v_{\left( {i,{\mathrm{min}}} \right)} - v_{\left( {i,{\mathrm{max}}} \right)}} \right)$$

In Eq. 2, *v*_*(i,*min)_ and *v*_*(i,*max)_ represent feasible flux range in each reaction from an unaltered model system, identified using flux variability analysis (FVA). For estimation of fluxes, these models were constrained using exchange rate for twenty-one metabolites including glucose, lactate and amino acids. (Supplementary File 5) Additional constraints on biomass reaction using experimental growth rates, ATP maintenance reaction as 1.07 mmol/gDW/h based on measurements^32^ and on oxygen uptake rate (OUR) based on estimations following flux balance estimations for maximal OUR feasible by the models. These contextualized models were evaluated following their in silico phenotypic predictions using constraints-based methods such as, flux balance analysis and uniform random flux sampling of flux solution space. We also added an in silico pseudo-hypoxia reaction (NAD [c] + H_2_[c] → NADH [c] + H[c]) involving the conversion of cytosolic NADH to NAD^+^ according to the previous study of testing the pseudo-hypoxia in cancer cells^39^, to estimate the levels of NADH and NAD^+^ in the cell-specific contextualized models.

### Flux variability analysis (FVA)

Plurality of solutions exists for the FBA problem, since the cell can choose multiple flux distributions to result in a unique objective function. FVA identifies the set of feasible fluxes at the optimal objective. The method calculates the minimum and maximum allowable fluxes through each reaction using a double optimization linear programming approach for each reaction of interest. The FVA problem, an extension of the FBA, is set up as

5 $${\mathrm{max}}/{\mathrm{min}}\left\{ {v_i} \right\}$$

Subject to,

6 $$S.v = 0$$

7 $$v_{{\mathrm{obj}}} \ge {\it{{\Bbb Y}Z}}_0$$

8 $$v_i^{{\mathrm{min}}} \le v_i \le v_i^{{\mathrm{max}}}$$

where *v*_obj_ is an optimal solution for (Eq. 1). ${\it{{\Bbb Y}}}$ is a control parameter to define the problem with respect to the default optimal state (${\it{{\Bbb Y}}}$ = **1**) or alternate sub-optimal network states (0 ≤ ${\Bbb Y}$ < **1**) for objective function. The non-uniqueness of the FBA solution allows calculation of a range of flux that is feasible for each reaction, thus defining the rigidity and plasticity of the network.

### Uniform random sampling of reaction flux

Similar to FVA, properties of metabolic flux states can be deciphered by random sampling of feasible flux space within the enclosing parallelepiped solution space^38^. This can be achieved by choosing a random point uniformly along each edge of parallelepiped following Monte Carlo sampling. Equation 3 illustrates how random points are chosen within the solution space.

9 $$\bf \alpha _i = \alpha _{i,{\mathrm{min}}}+{R_n}\left( {{{\alpha}} _{i,{\mathrm{max}}} - {{\alpha}} _{i,{\mathrm{min}}}} \right)$$

In Eq. 3, ***Rn*** is a random number chosen between 0 and 1 while *α*_i,min_ and *α*_i,max_ defines the flux range of feasible flux state along each reaction vector identified using FVA. These points can then be further compared to the set of constraints imposed on a constrained based metabolic model, in order to verify whether the random point falls in solution space.

Solution sampling in this manner not only offers insights about plasticity of the metabolic network but offers latent information about metabolic flux states such as, coregulated list of trans-acting metabolic reactions. In addition, information about rewiring of the metabolic network imposed by system specific constraints can also be elucidated by inspecting the population distribution of random sampling for each reaction^38^.

Flux sampling for U87MG and NSP cell line models was carried out using a Markov Chain Monte Carlo method of Artificial Centering Hit-and-Run (ACHR) sampler from COBRA toolbox. The initial point for the sampler was chosen amongst 1000 warmup points identified by combining random and orthogonal points. A total of 50,000 randomly distributed sampling points were computed with 1000 iterations between each stored point. Distribution of individual reaction flux values across the sampling population was represented as a histogram of feasible flux value and associated frequency in the convex polytope of solution space.

### Reporting summary

Further information on experimental design is available in the Nature Research Reporting Summary linked to this article.

## Supplementary information

Supplementary File

Supplementary File 2

Supplementary File 3

Supplementary File 4

Supplementary File 5

Reporting Summary

## Data Availability

The whole exome sequencing data for U87MG and NSP cells from this study is deposited in Sequence Read Archive (SRA) submission: SUB3253007, with accession id: SRR7091404 (U87MG) and SRR7091405 (NSP).
