# Supplementary material for: Integrated genetic and metabolic landscapes predict vulnerabilities of temozolomide resistant glioblastoma cells
Source: NPJ Syst Biol Appl. 2021 Jan 8;7:2. doi: 10.1038/s41540-020-00161-7 (PMC7794364; doi:10.1038/s41540-020-00161-7)
Supplement: Supplementary file 1 — Supplementary File [file 41540_2020_161_MOESM1_ESM.pdf]

Supplementary Figures 1 to 21 and

Supplementary Tables 1 to 4

Integrated Genetic and Metabolic Landscapes Predict Vulnerabilities Of Temozolomide Resistant Glioblastoma Cells

Selva Rupa Christinal Immanuel[1,2], Avinash D. Ghanate[1,2], Dharmeshkumar S Parmar[1,2], Ritu Yadav[1,2], Riya Uthup[1], Venkateswarlu Panchagnula[1,2] and Anu Raghunathan[1,2*]

[1]Chemical Engineering Division, CSIR-National Chemical Laboratory, Pune, India.
[2]Academy of Scientific and Innovative Research (AcSIR), CSIR-National Chemical Laboratory, Pune, India.

*Corresponding author:
Dr. Anu Raghunathan,
Principal Scientist, Biochemical Engineering unit, Chemical Engineering division,
CSIR, National Chemical Laboratory (CSIR-NCL), Pune. India.
E-mail: anu.raghunathan@ncl.res.in
Tel: +91-20-2590-3067

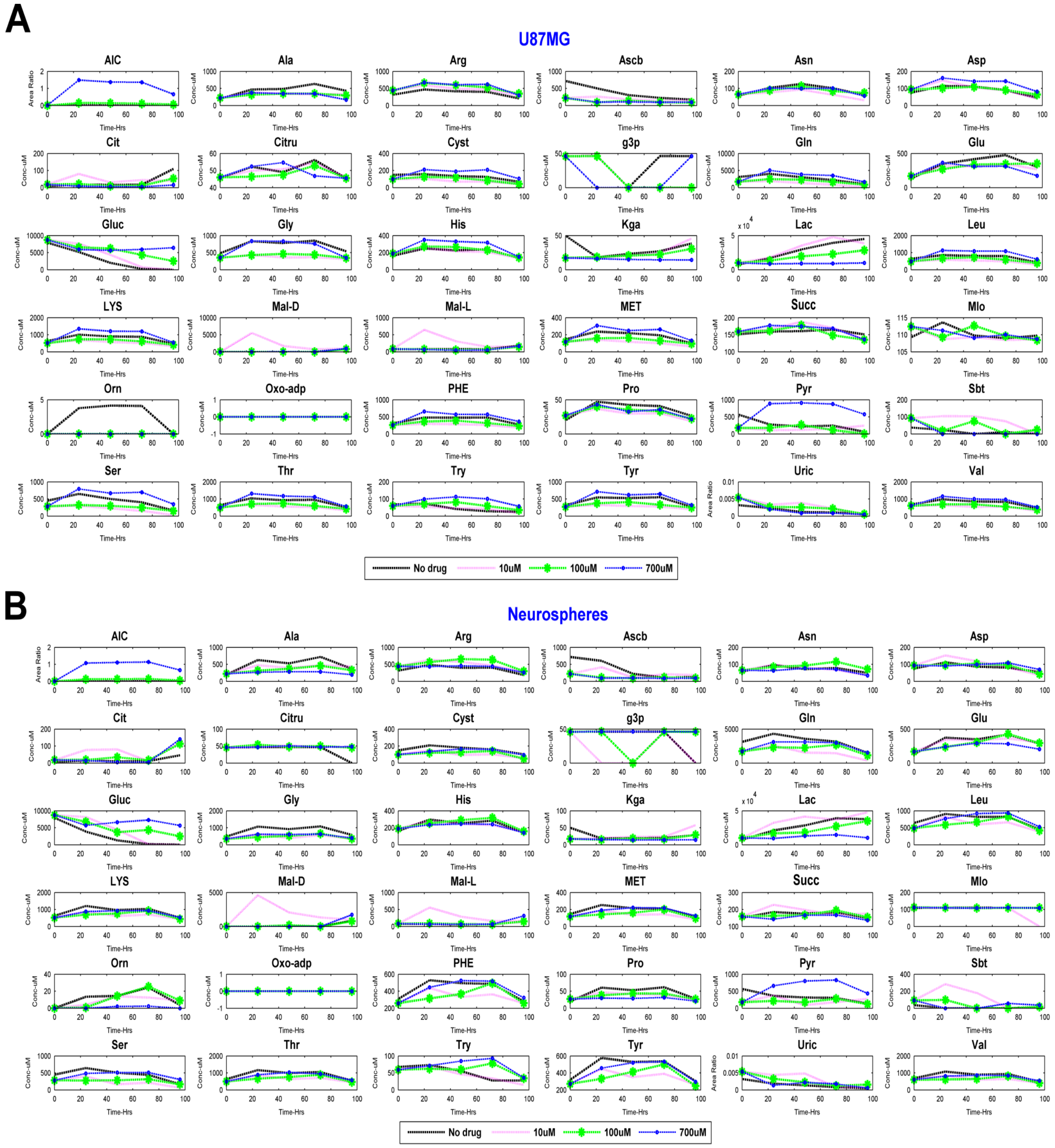

Supplementary Fig. 1. Extracellular absolute quantification values from LCMS-MS analysis. The plots indicate profiles over time. **A.** U87MG and **B.** NSP cells.

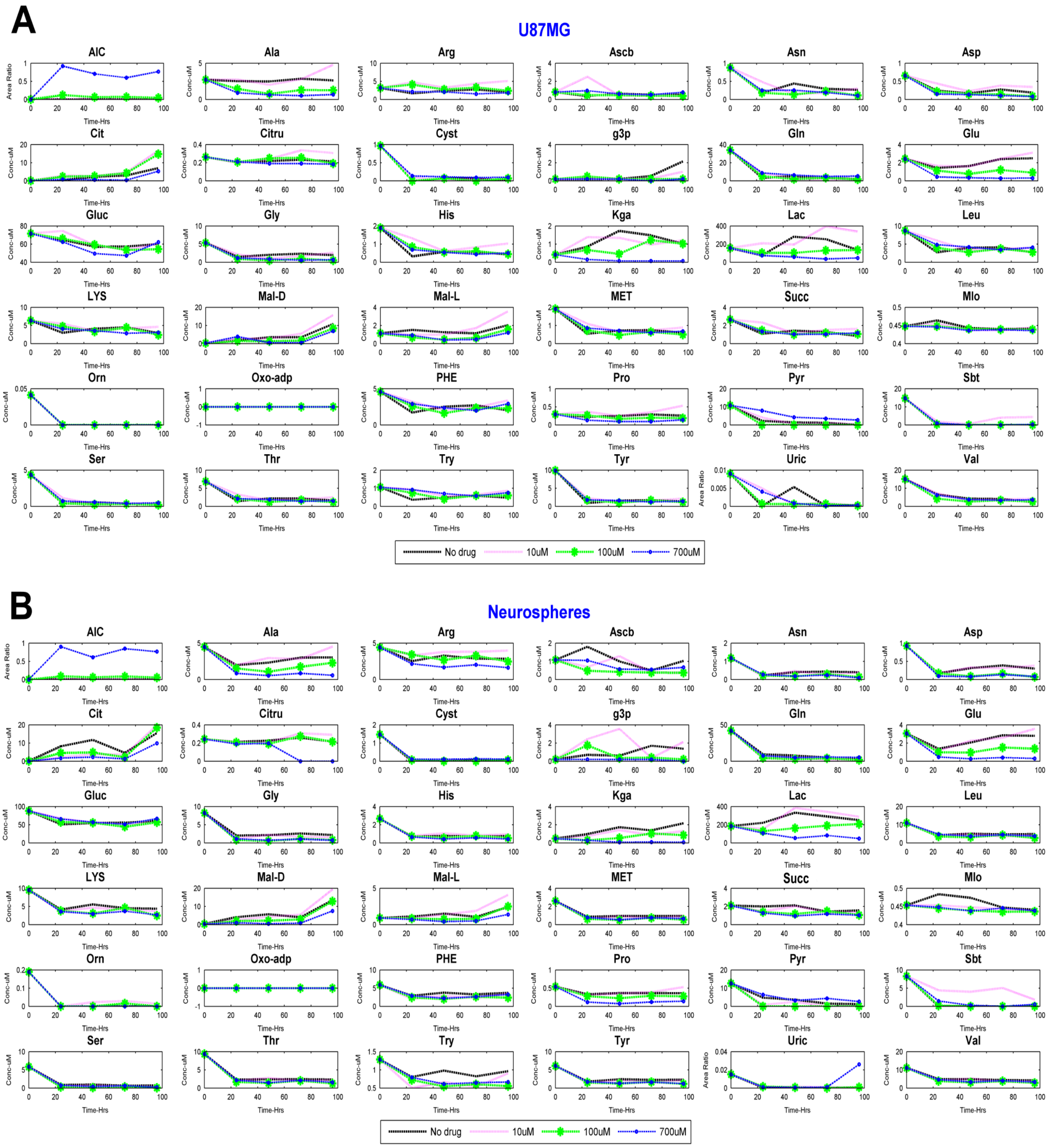

Supplementary Fig. 2. Intracellular absolute quantification values from LCMS-MS analysis. The plots indicate profiles over time. **A.** U87MG and **B.** NSP cells.

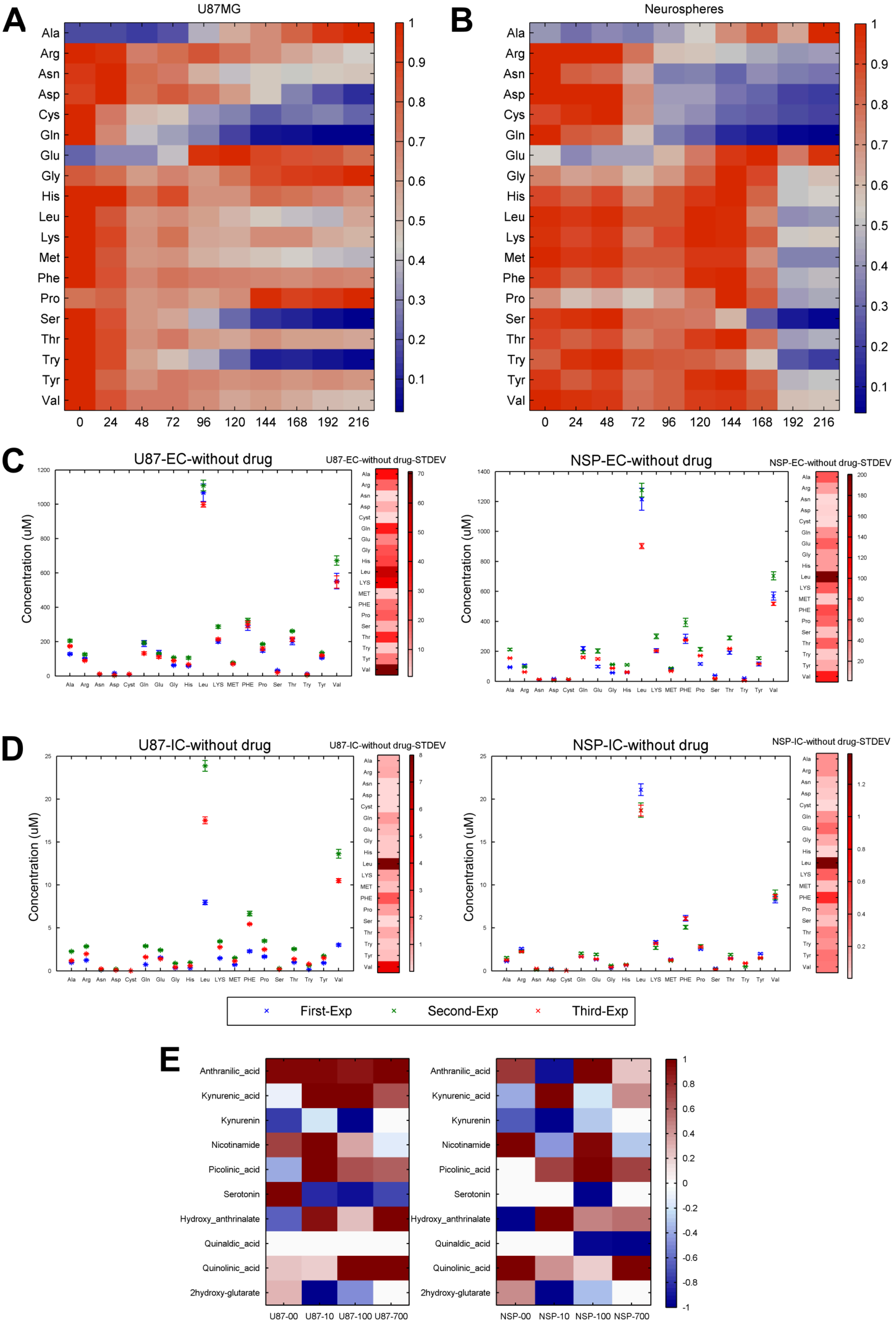

Supplementary Fig. 3. LCMS-MS analysis using sampling method. Heatmap indicate absolute quantification profiles over time. **A.** U87MG and **B.** NSP cells. **C and D** are plotted to show the recovery across three biological replicates.

AKG concentration

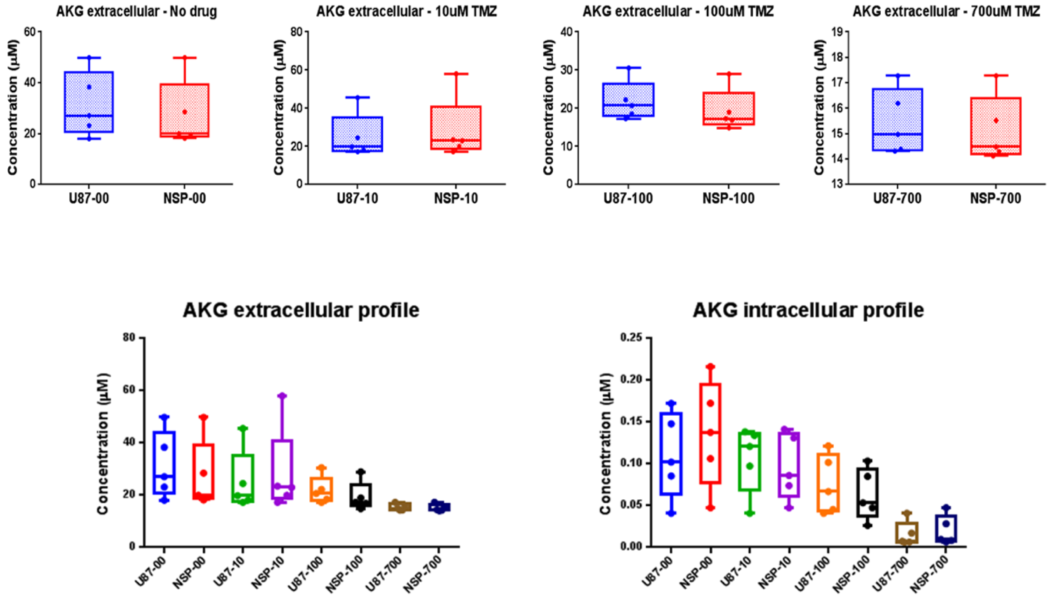

Supplementary Fig. 4. LCMS-MS analysis of AKG concentration in extracellular and intracellular profiles of U87MG and NSP at different concentrations of temozolomide.

Succinate concentration

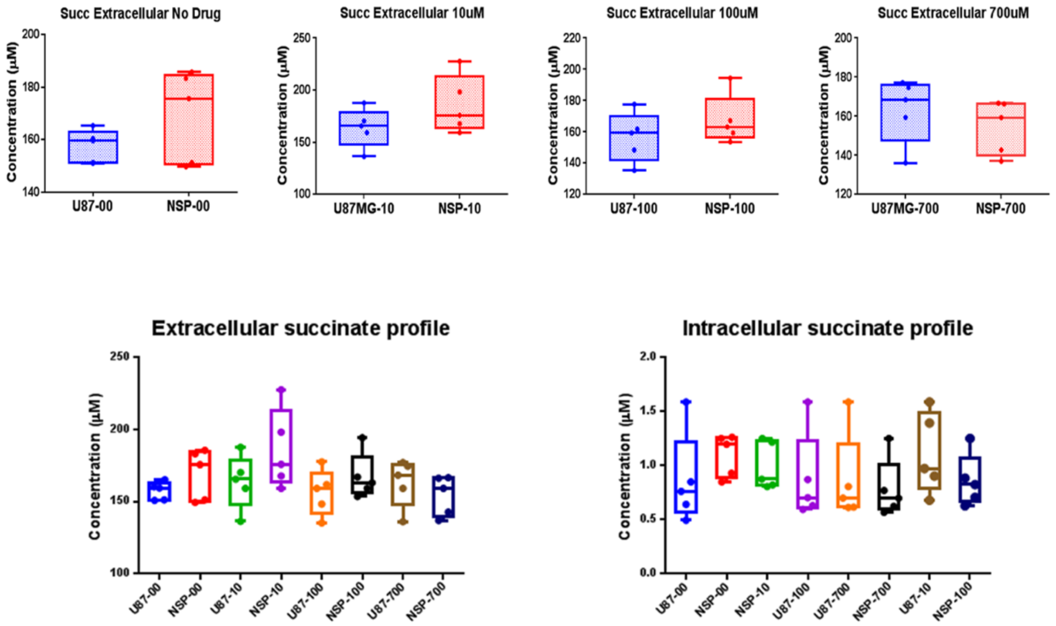

Supplementary Fig. 5. LCMS-MS analysis of succinate concentration in extracellular and intracellular profiles of U87MG and NSP at different concentrations of temozolomide.

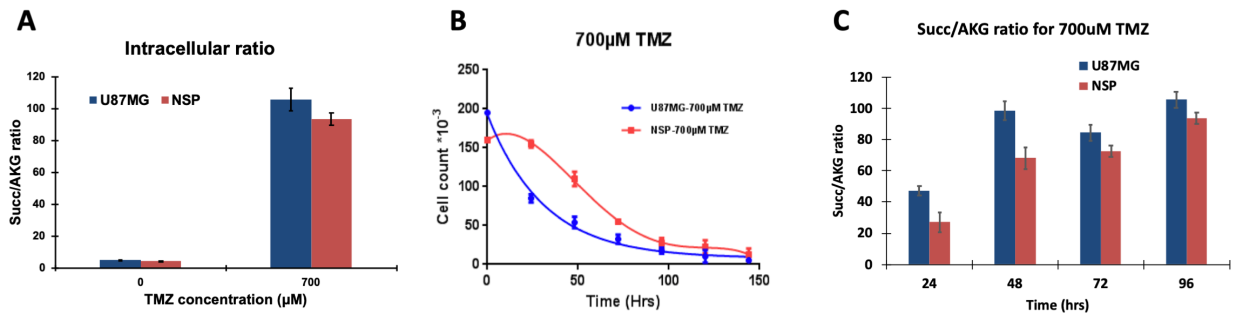

Supplementary Fig. 6. Succinate/AKG ratios. A. Intracellular profiles of Succ/AKG ratio for no drug versus 700 uM concentration of TMZ **B.** Growth profile at 700 uM TMZ. **C.** All time point intracellular profiles of Succ/AKG ratio at 700 uM concentration of TMZ.

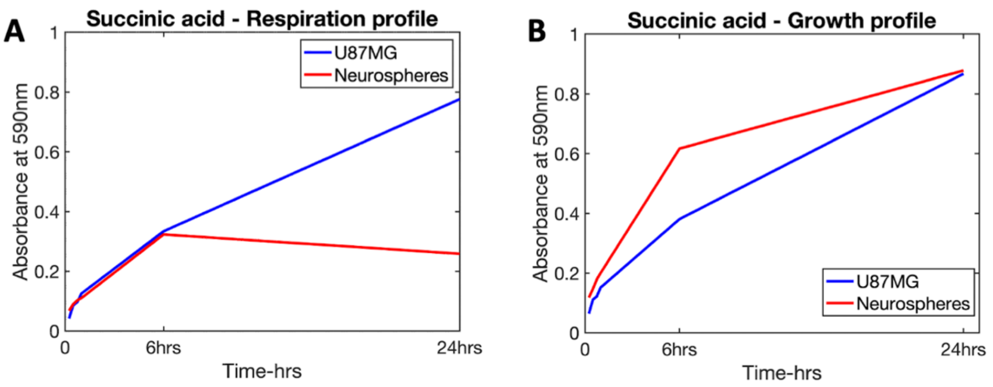

Supplementary Fig. 7. Succinate BIOLOG profiles. A. Respiration profiles and B. Growth profiles for U87MG and NSP in the presence of succinate as sole carbon/nitrogen source.

A

Features	U87MG Exome	NSP Exome
Targets with base coverage at 1x	98.80%	98.64%
Targets with base coverage at 20x	92.22%	95.00%
Targets with base coverage at 100x	18.60%	27.08%
Targets with base coverage at 500x	0.11%	0.16%
Targets with no strand bias	92.88%	94.91%
Targets with full coverage	96.95%	97.43%
Bases in target region	46208266	46208266

B

U87MG-annotated variants	
Total no. of variants	30704
SNP (97%)	29668
INDELs (3%) (No MNPs)	1036

C

Neurospheres-annotated variants	
Total no. of variants	31776
SNP (96%)	30575
INDELs (4%) (including 12 MNPs)	1201

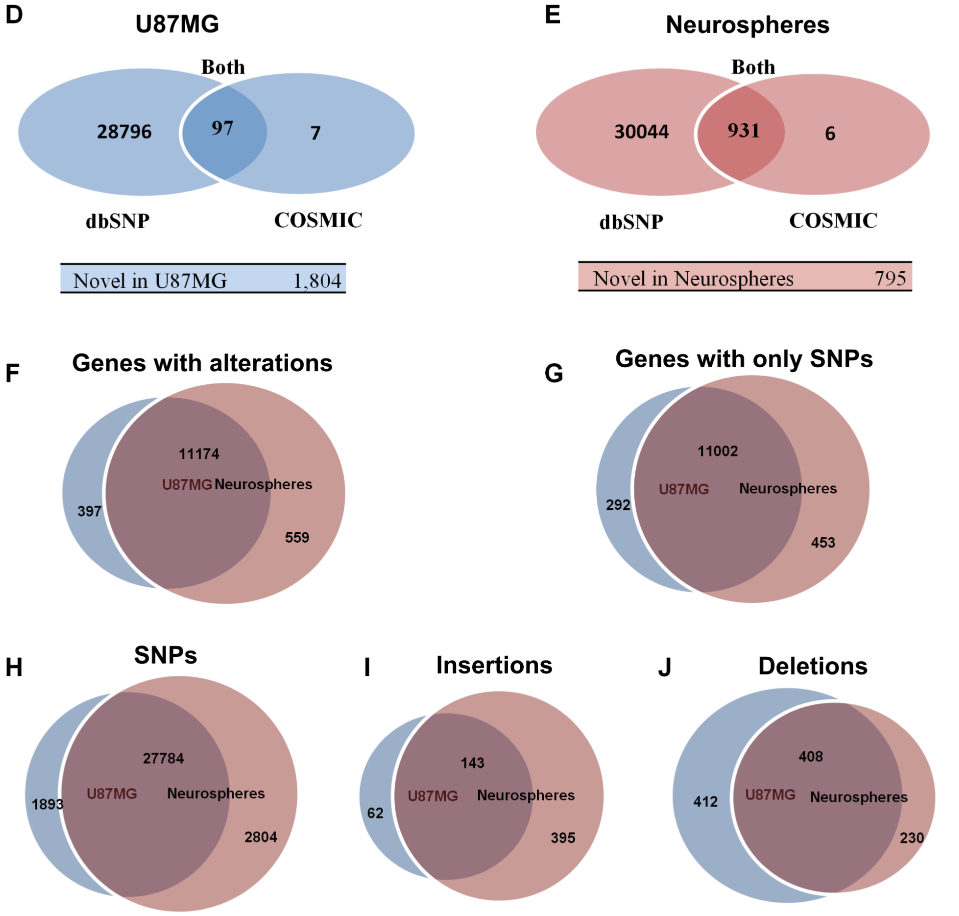

Supplementary Fig. 8. Statistical analysis of Exome sequencing data. A. Coverage from the Exome analysis. **B and C.** Number of annotated variants in U87MG and NSP. **D and E.** Concordance analysis for mutations available in COSMIC and dbSNP databases for U87MG and NSP. Common and unique analysis of mutations across U87MG and NSP for all genes (**F**), genes with SNPs (**G**), SNPs (**H**), Insertions (**I**) and Deletions (**J**).

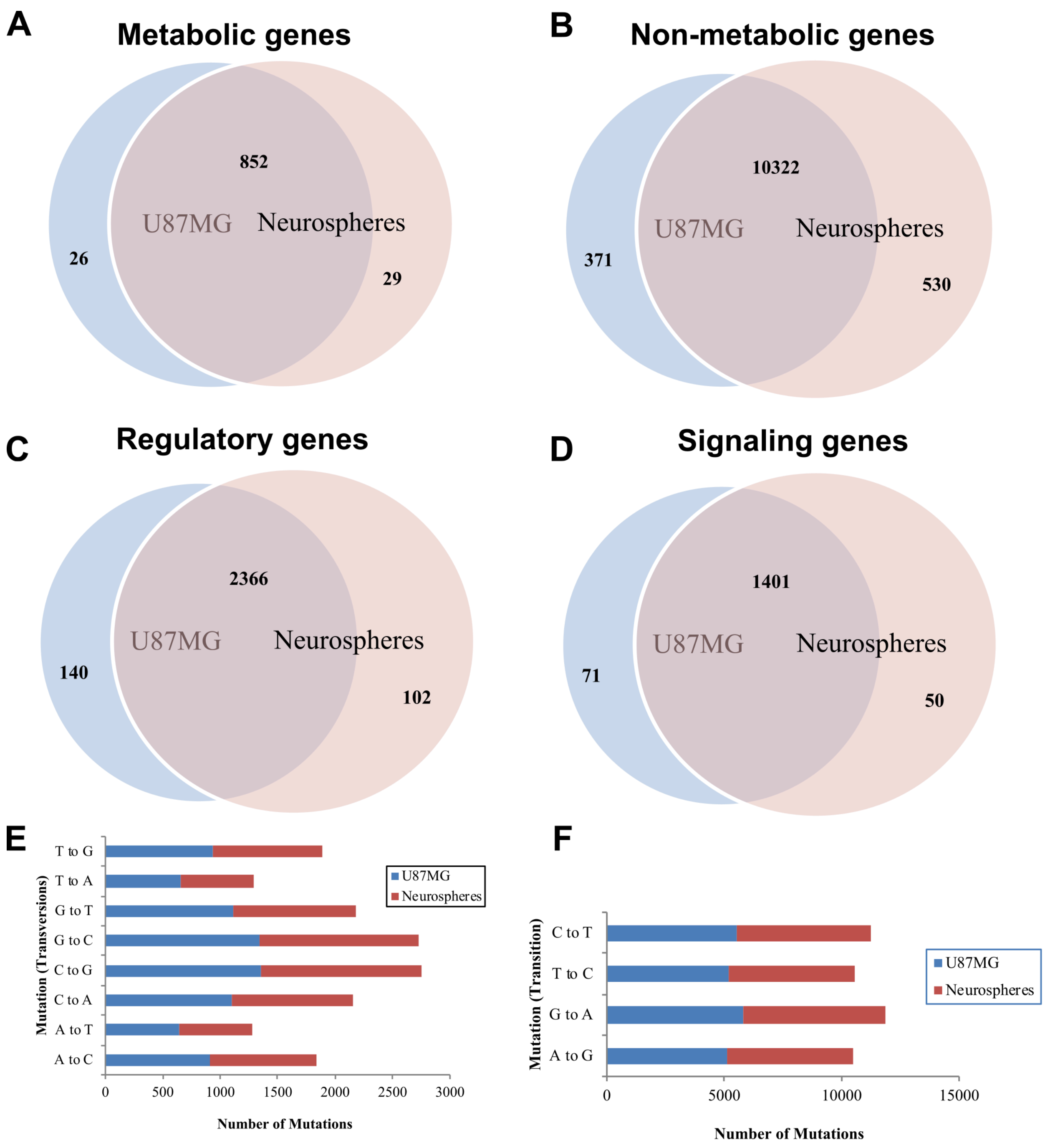

Supplementary Fig. 9. Functional characterization of genes using Exome data. Common and unique analysis of mutations across U87MG and NSP for metabolic (A), non-metabolic (B), regulatory (C) and signaling (D) genes. Transversions (E) and transitions (F) across U87MG and NSP cells identified using Exome sequencing.

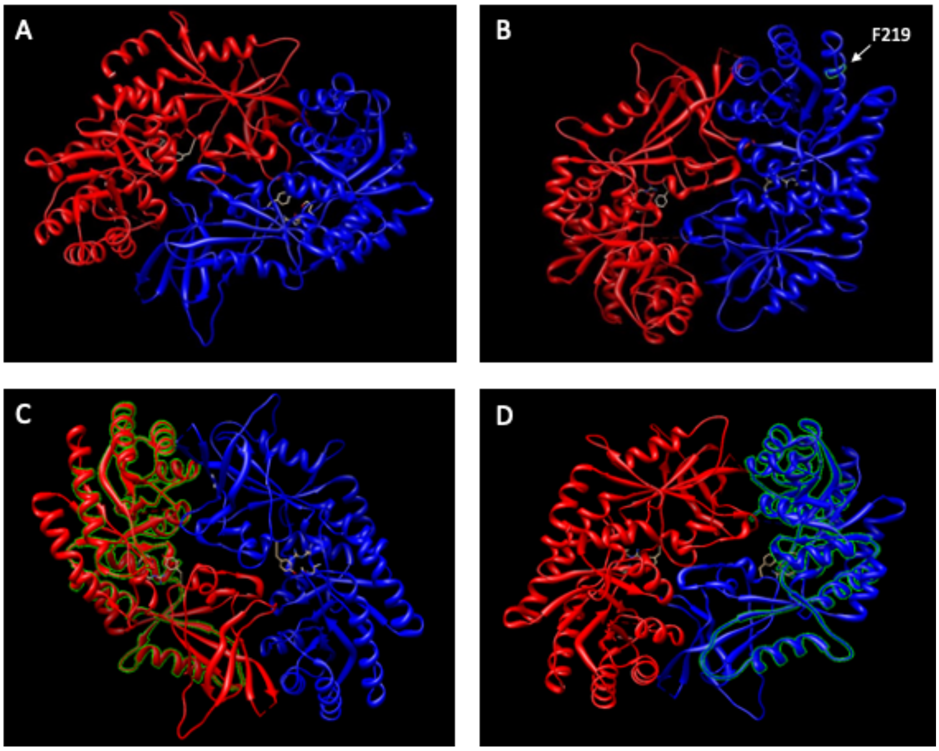

Supplementary Fig. 10. Structural changes due to a deletion in the ODC1 gene. A. Structure of ODC1 enzyme. **B.** The position of deletion has been highlighted (Chr 2 #10583624 that changes from CA ---> C, resulting in a protein change of p.F219fs (Frameshift). **C and D** shows the effect of frameshift deletion on the ODC1 monomers (highlighted in green are the affected regions). This changes ornithine production and excretion profile between U87MG and NSP extracellularly.

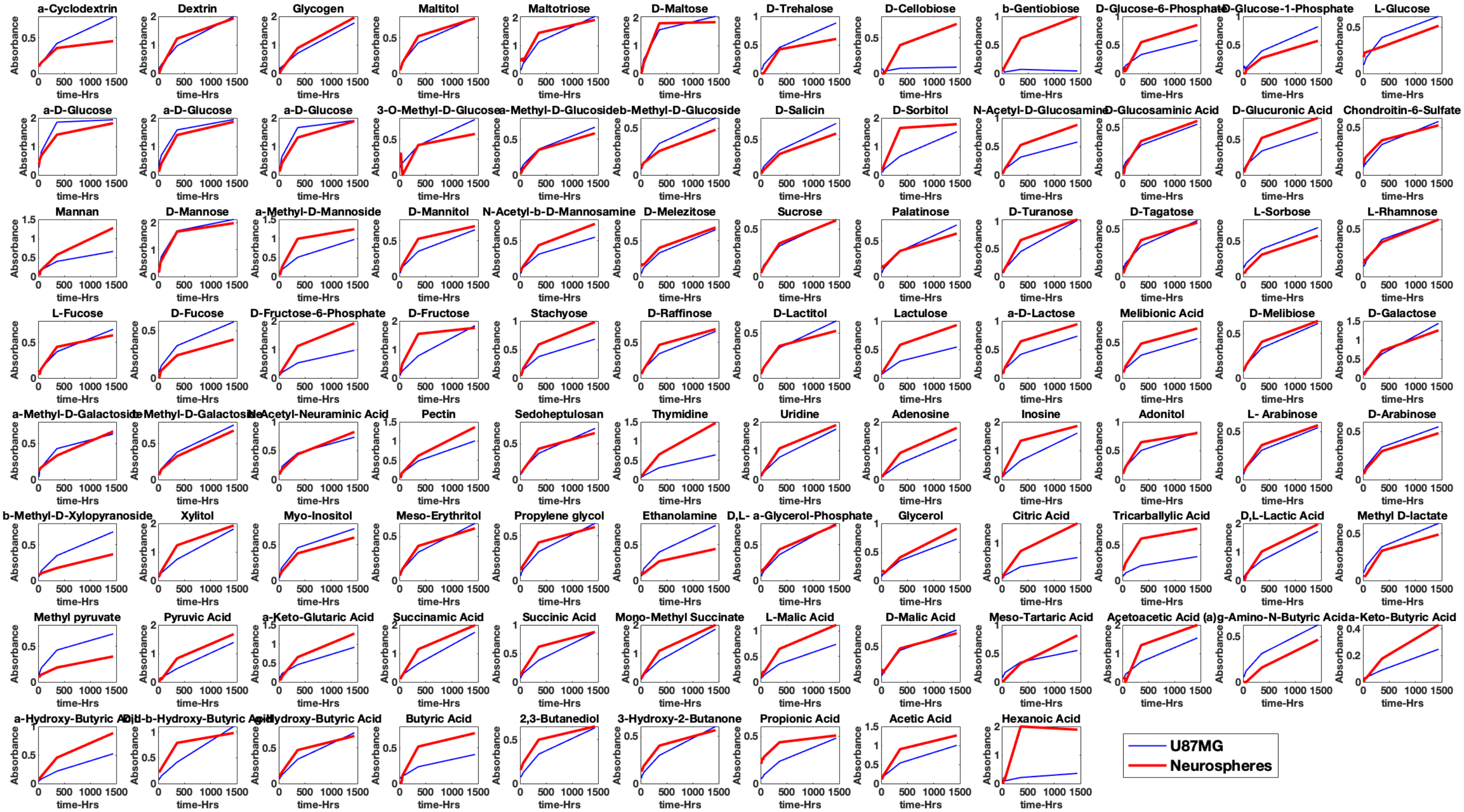

Supplementary Fig. 11. Growth of U87MG (blue) and NSP (red) on BIOLOG PMM plate 1 substrates.

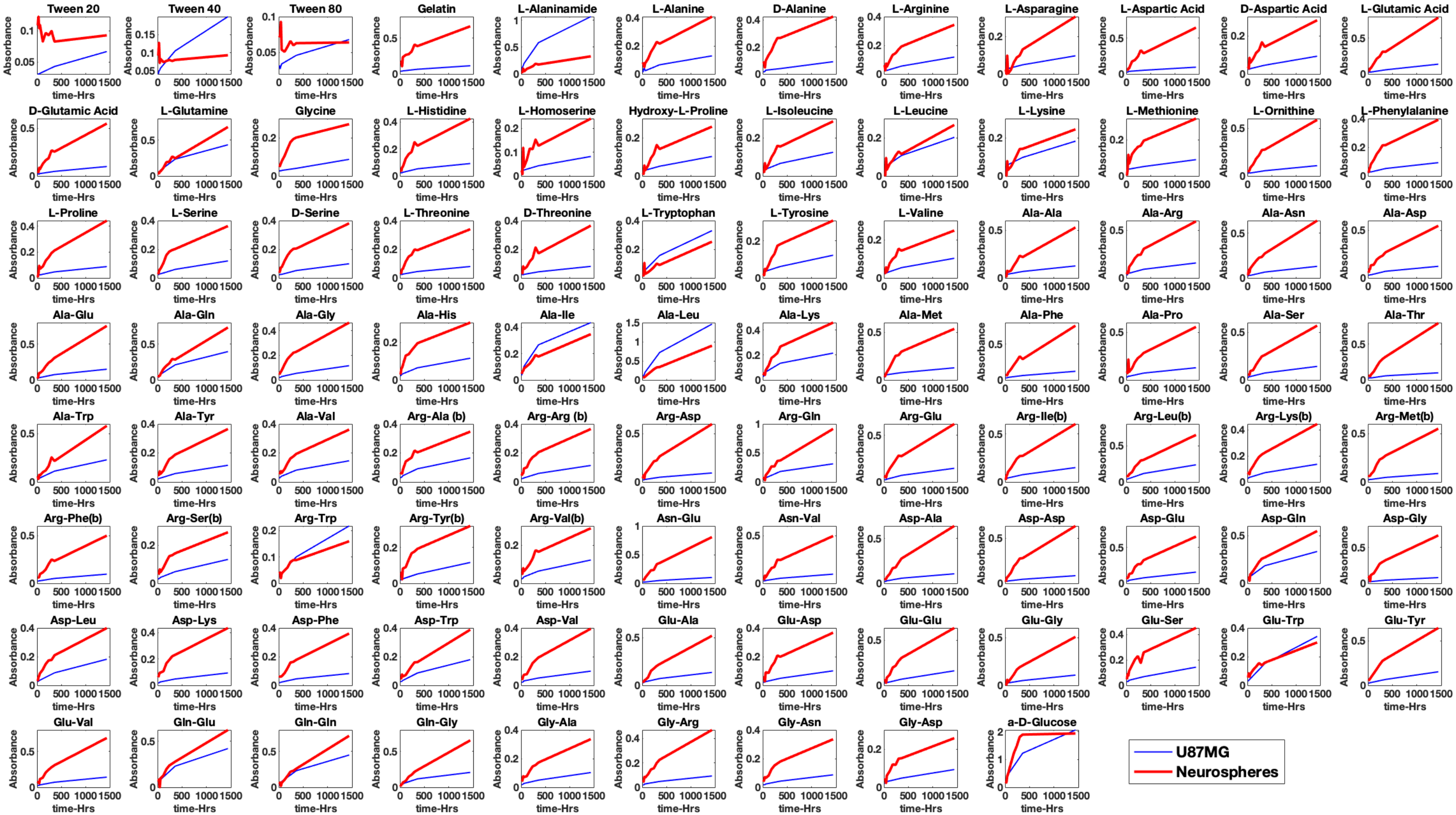

Supplementary Fig. 12. Growth of U87MG (blue) and NSP (red) on BIOLOG PMM plate 2 substrates.

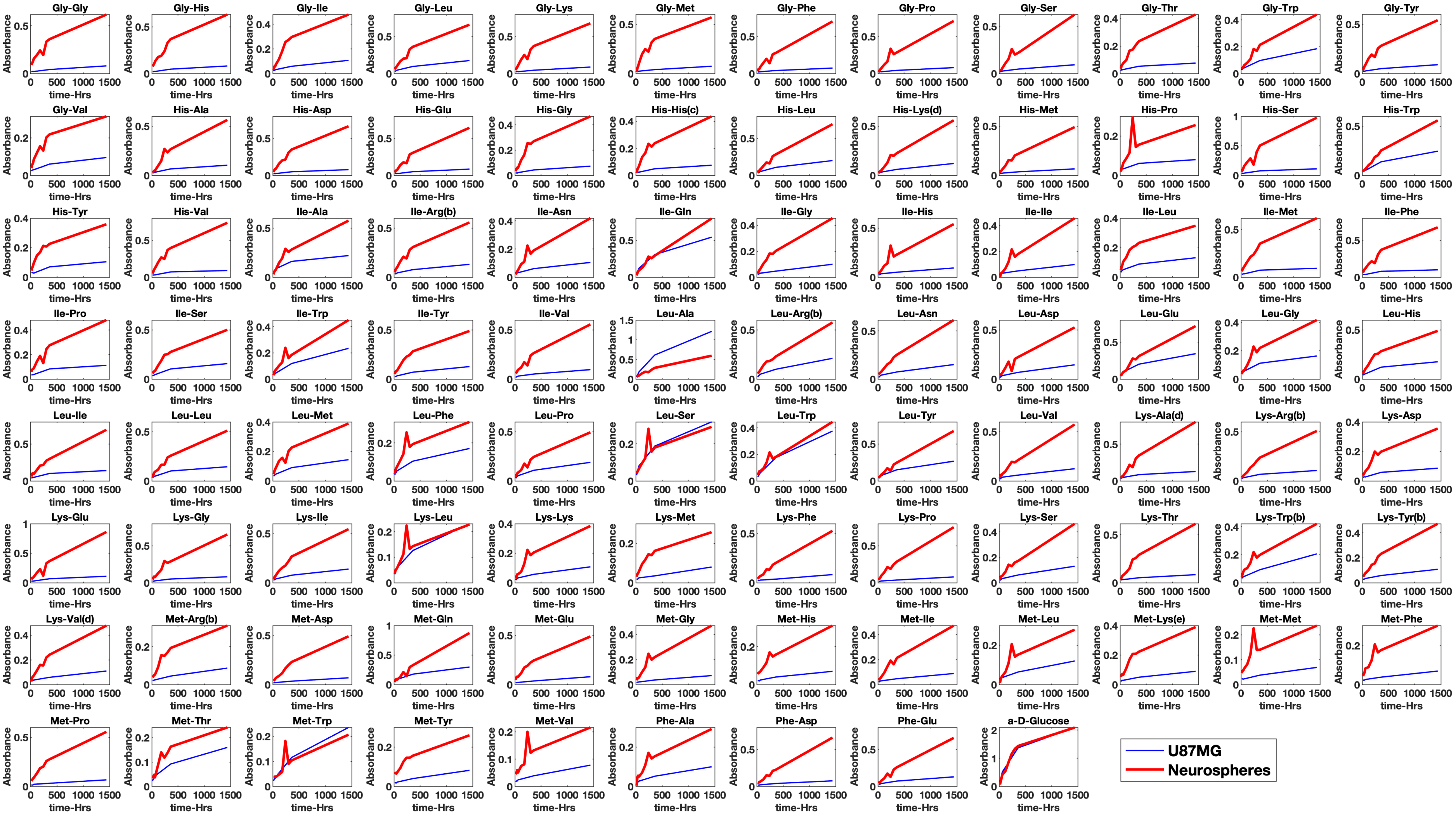

Supplementary Fig. 13. Growth of U87MG (blue) and NSP (red) on BIOLOG PMM plate 3 substrates.

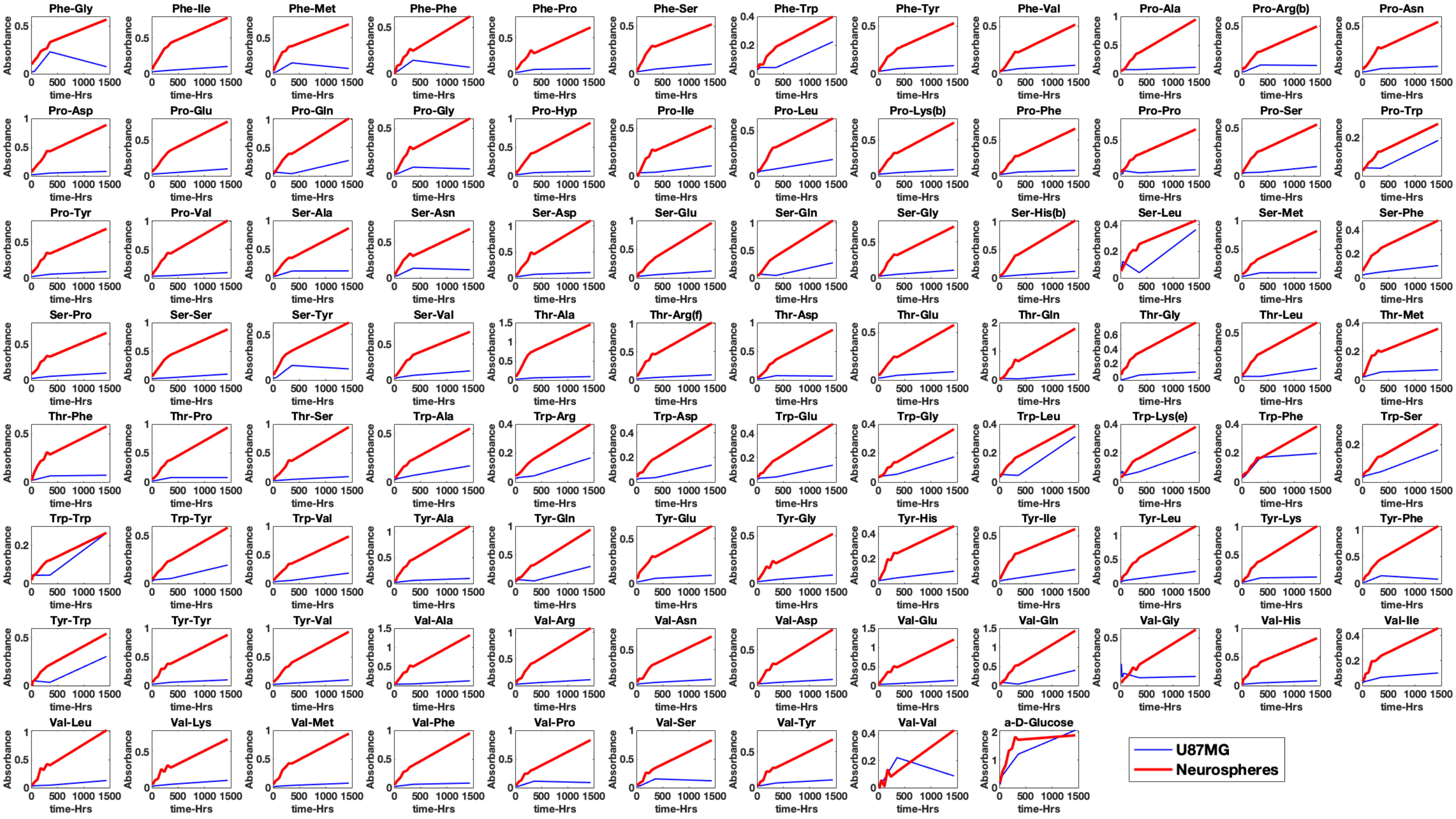

Supplementary Fig. 14. Growth of U87MG (blue) and NSP (red) on BIOLOG PMM plate 4 substrates.

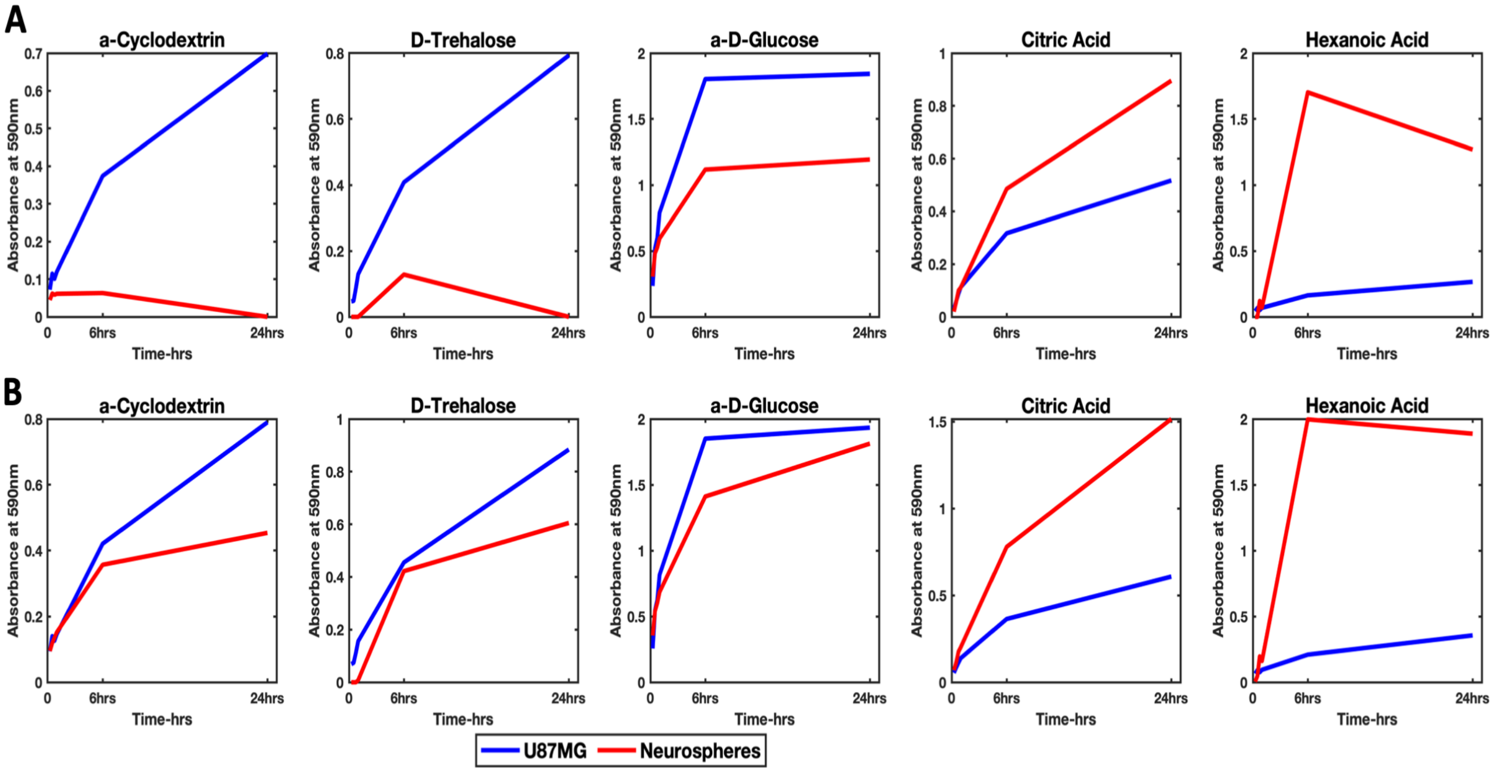

Supplementary Fig. 15. A. Respiration and B. Growth of U87MG (blue) and NSP (red) on BIOLOG PMM plate selected substrates.

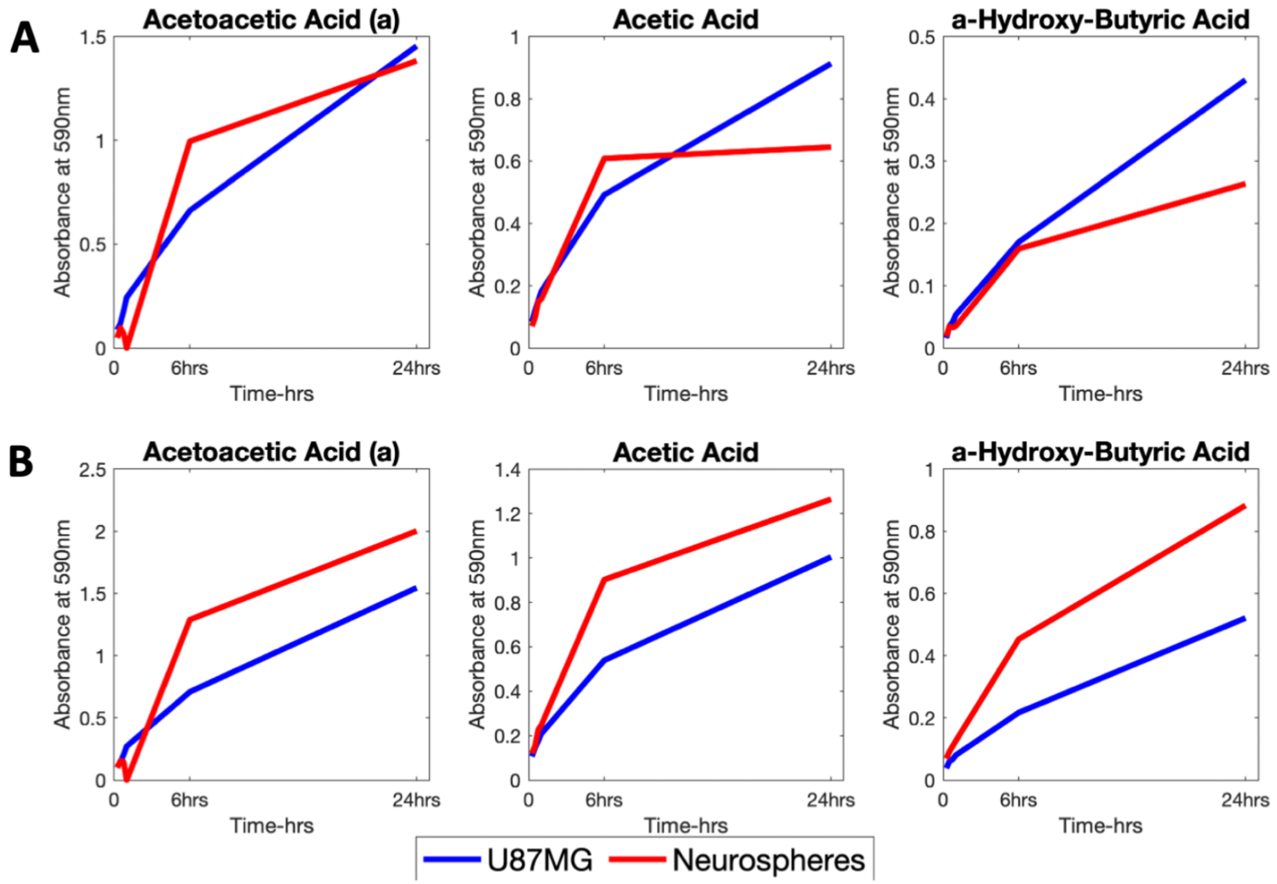

Supplementary Fig. 16. BIOLOG profiles for A. Respiration and B. Growth on selected cholesterol related substrates.

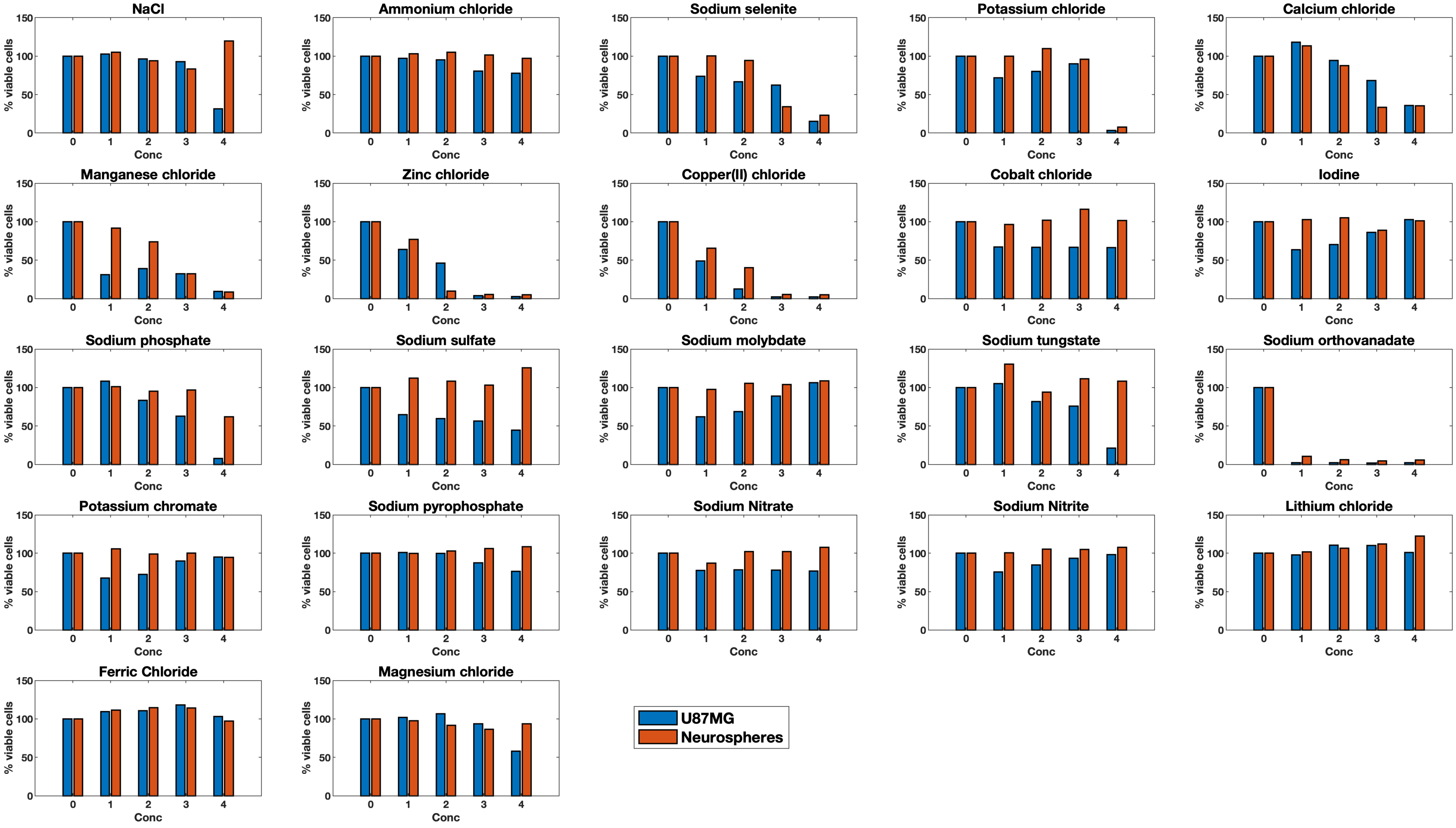

Supplementary Fig. 17. Growth of U87MG (blue) and NSP (red) on BIOLOG PMM plate 5 substrates (Ions).

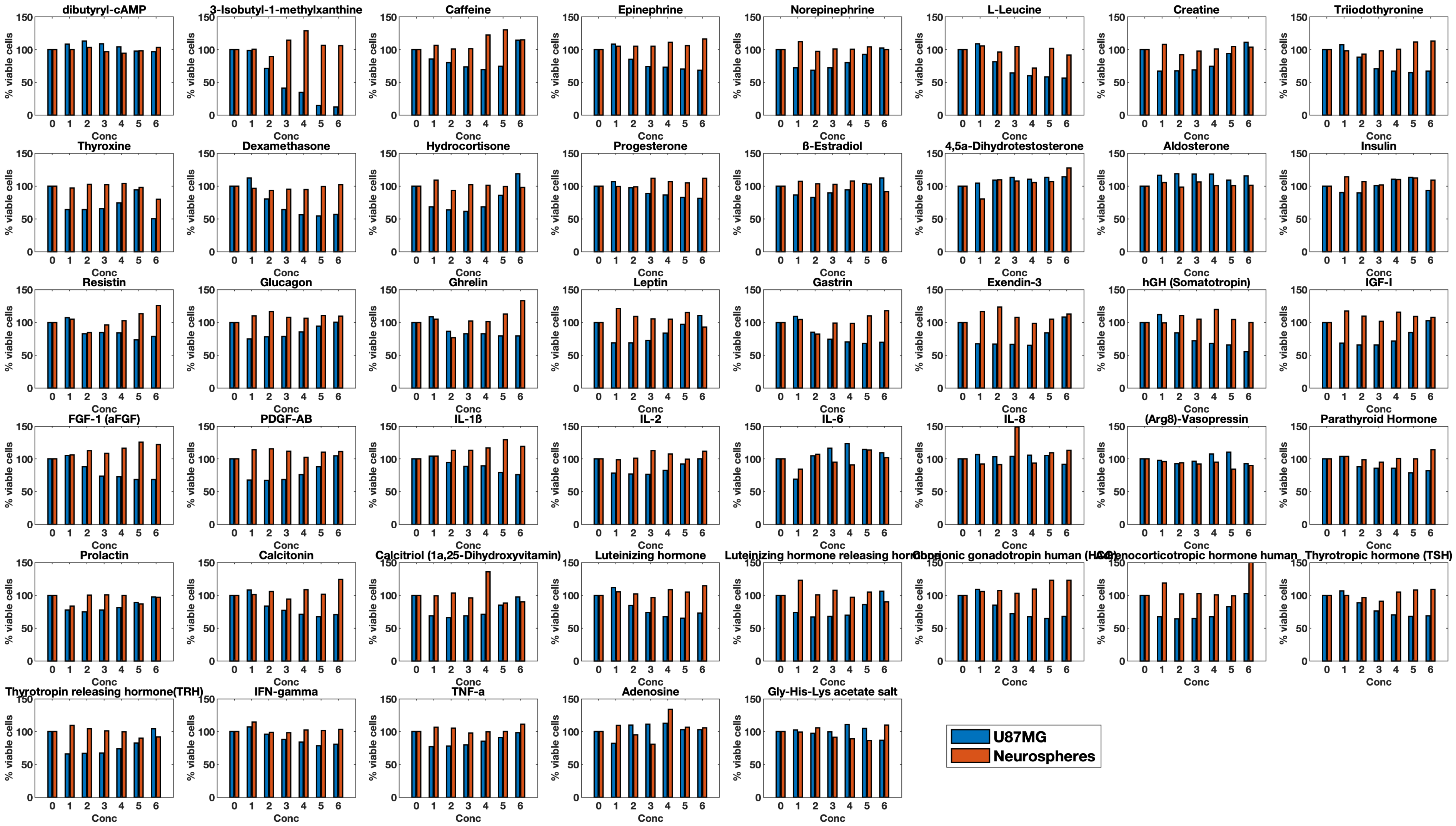

Supplementary Fig. 18. Growth of U87MG (blue) and NSP (red) on BIOLOG PMM plate 6 to 8 substrates (Hormones and Cytokines).

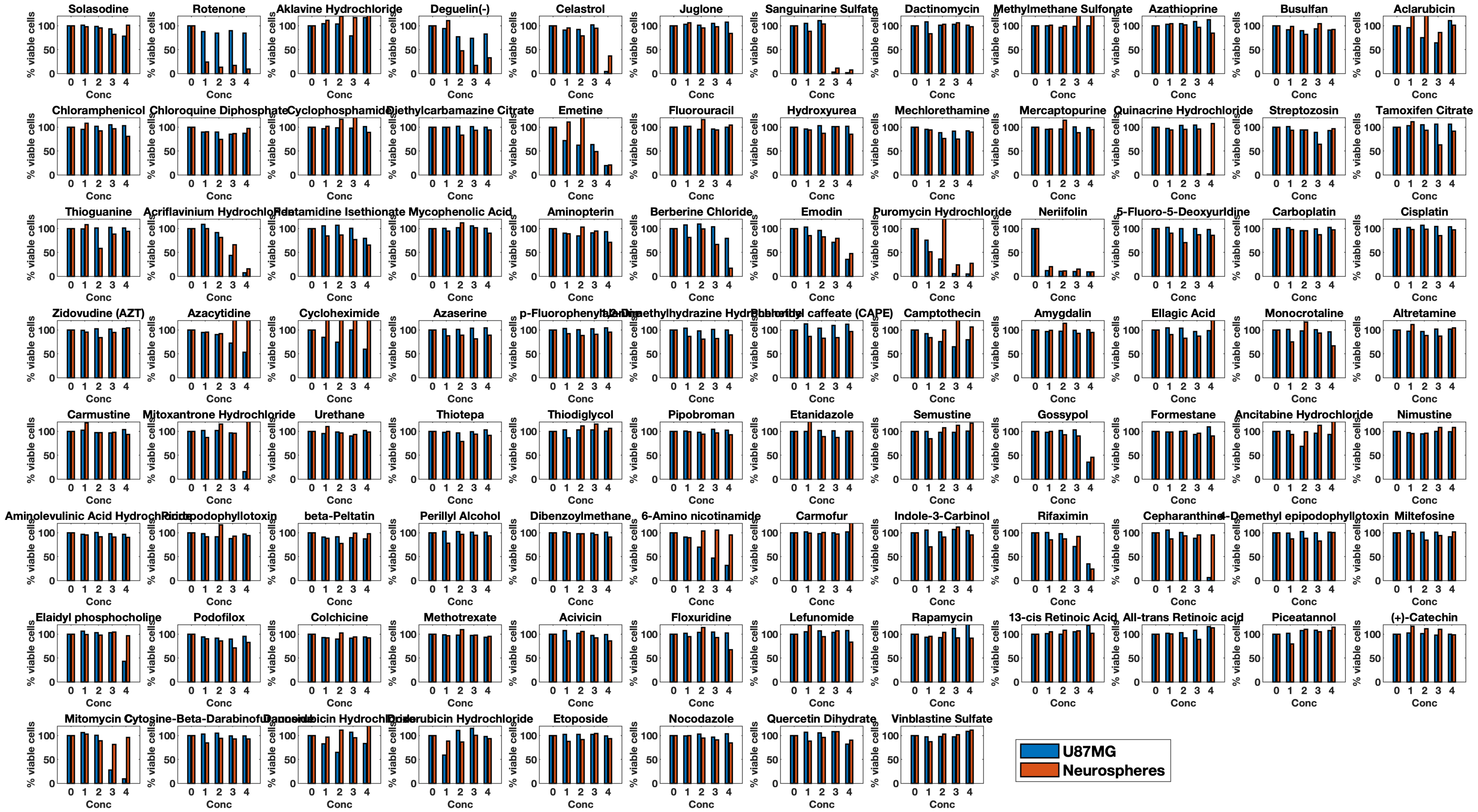

Supplementary Fig. 19. Growth of U87MG (blue) and NSP (red) on BIOLOG PMM plate 11 to 14 (Drug panels).

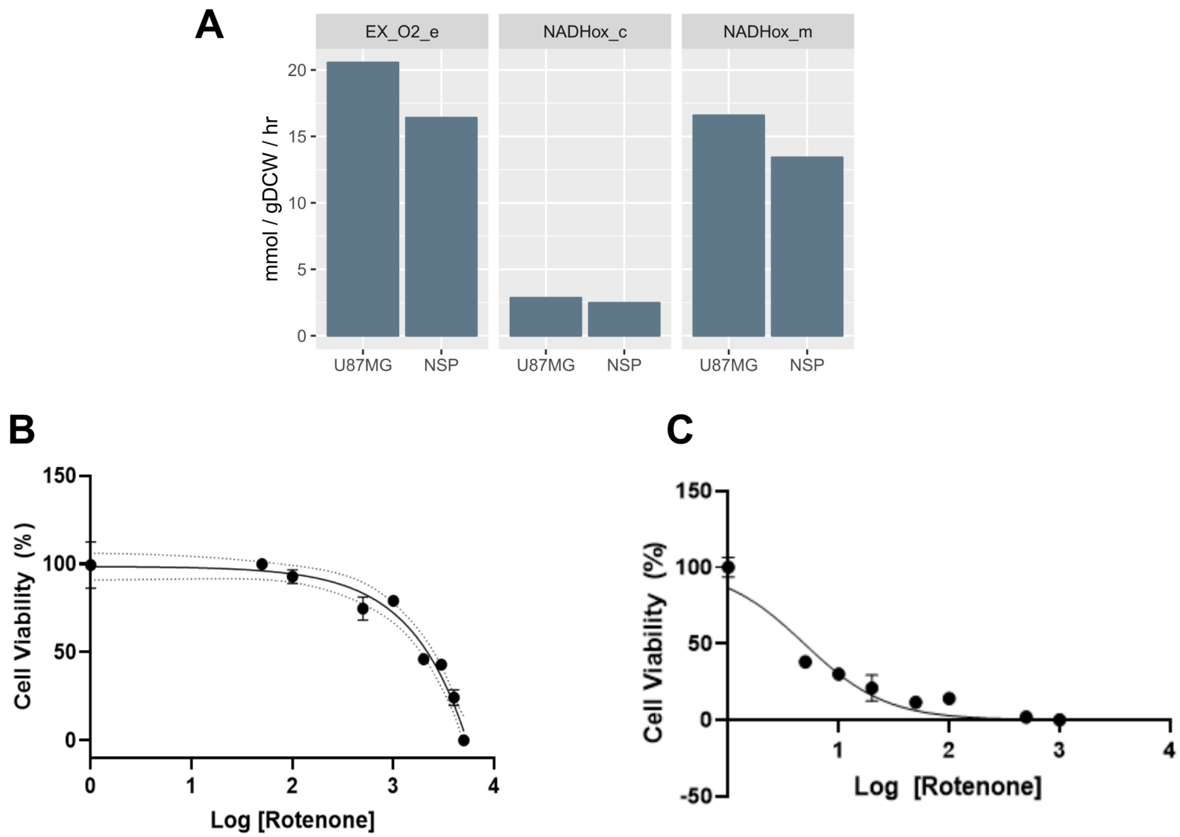

Supplementary Fig. 20. Model predictions and rotenone IC50. A. NADH oxidase predictions from in silico metabolic model. Rotenone IC50 curve for U87MG (**B**) and NSP (**C**)

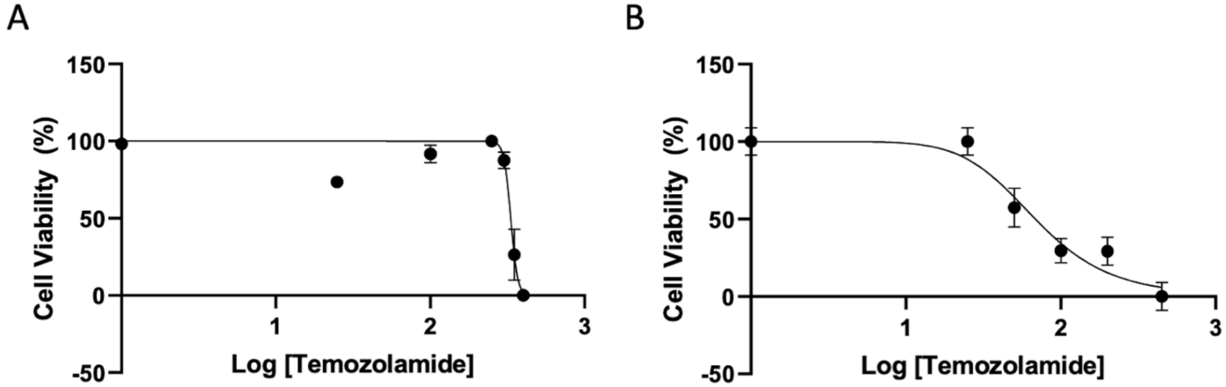

Supplementary Fig. 21. Sensitivity assay in the presence of 5nM rotenone for U87MG and NSP cells. A. IC50 of temozolomide in U87MG cells in the presence of rotenone - 331.8μM. **B.** IC50 of temozolomide in the presence of rotenone - 1.8μM.

Gene Sym	Type	Variant_Classification	dbSNP_RS	Genome Change	Protein Change	PPH2_Class	PPH2_Proba	PPH2_FDR
EXTL1	SNP	Missense	rs2736831	g.chr1:26357656C>A	p.H379N	deleterious	0.952	0.161
PGM1	SNP	Missense	rs1126728	g.chr1:64097432C>T	p.R221C	deleterious	1	0
RHBG	SNP	Missense	rs2245623	g.chr1:156347131G>A	p.G76D	deleterious	0.998	0.0443
FMO2	SNP	Missense	rs2020862	g.chr1:171168584C>T	p.S195L	deleterious	0.994	0.114
PIGC	SNP	Missense	rs1063412	g.chr1:172410967G>A	p.P266S	deleterious	0.999	0.0222
EPHX1	SNP	Missense	rs1051740	g.chr1:226019633T>C	p.Y113H	deleterious	0.997	0.0665
PLCL1	SNP	Missense	rs1064213	g.chr2:198950240G>A	p.V667I	deleterious	0.974	0.15
GLB1	SNP	Missense	rs7637099	g.chr3:33138549G>A	p.P10L	deleterious	0.982	0.143
INMT	SNP	Missense	rs4720015	g.chr7:30795436T>G	p.F254C	deleterious	0.999	0.0222
SLC29A3	SNP	Missense	rs2277257	g.chr10:73082563A>G	p.R18G	deleterious	0.996	0.0887
DLAT	SNP	Missense	rs10891314	g.chr11:111916647G>A	p.D451N	deleterious	0.997	0.0665
PFAS	SNP	Missense	rs4791641	g.chr17:8161149C>T	p.P367L	deleterious	0.96	0.157
GUCY2F	SNP	Missense	rs12008095	g.chrX:108708552A>G	p.L284P	deleterious	0.991	0.123
NSP unique mutations								
SLCA15	SNP	Missense	rs3027956	g.chr19:47291174G>C	p.P17A	neutral	0.47	0.234
ATP4A	SNP	Missense	rs2733743	g.chr19:36050969A>G	p.V265A	neutral	0	0.665
ACO1	SNP	Missense	rs147879556	g.chr9:32430510C>A	p.P555H	deleterious	0.999	0.0222
U87MG unique mutations								
SDHA	SNP	Missense	rs76896145	g.chr5:236649C>T	p.S456L	deleterious	0.998	0.0443

Supplementary Table 1. PolyPhen predictions of selected deleterious mutations on function of proteins. Complete list of predictions is provided in Supplementary File 3.

Transporter	Exome data			Gene Expression		
	**NSP**	**U87MG**	**Type of alterations**	**NSP**	**U87MG**	**DWN/UP**
ABCA1	6	6	3 - Silent; 3 - Missense	1.51419968	1	Upregulation
ABCA2	6	5	2 - Silent; 1 - Missense; 1 - 5'Flank; 1 - 3'UTR; 1 - Silent (NSP)	2.04745466	1	Upregulation
ABCB10	1	1	1 - Intron	2.70171406	1	Upregulation
ABCB6	1	1	1 - Silent	1.42188687	1	Upregulation
ABCB8	3	3	2- Intron; 1 - Silent	1.12700196	1	Upregulation
ABCC1	2	2	2 - Silent	2.9996416	1	Upregulation
ABCC2	1	1	1 - Missense	6.86850362	1	Upregulation
ABCC3	2	2	2 - Intron	1.20588067	1	Upregulation
ABCC4	8	9	6 - Silent; 2 - 3'UTR; 1 - Silent (U87MG)	4.71982757	1	Upregulation
ABCC5	3	5	3 - Silent; 2 - Missense (U87MG)	12.5418868	1	Upregulation
ABCC9	2	1	1 - Silent; 1 - Intron (NSP)	2.56292428	1	Upregulation
ABCD3	1	1	1 - Silent	1.75919984	1	Upregulation

Supplementary Table 2. ABC transporters and their associated mutations, expression profiles for understanding mutational effects on expression of genes. Complete list of ABC transporters and their mutational details are provided in Supplementary File 4.

Model details	Biomass (Fixed)	In silico (mMol/gDCW)				Accuracy (%)
		Glucose	Glutamine	Tryptophan	Serine	
U87MG 0uM TMZ	0.0217	-0.71958	-0.20847	-0.00445	-0.02838	94%
U87MG 10uM TMZ	0.016	-0.64917	-0.10801	-0.00257	-0.01734	90%
NSP 0uM TMZ	0.0149	-0.73892	-0.16485	-0.00476	-0.02862	92%
NSP 10uM TMZ	0.0096	-0.87954	-0.13002	-0.00424	-0.02118	89%

Supplementary Table 3: Accuracy of prediction of uptake rates for four essential metabolites.

Cell	NADH (pmoles)	NAD+ (pmoles)	NAD+/NADH
U87MG	1.728	1.15	0.66
NSP	0.677	3.75	5.5

Supplementary Table 4: NADH/NAD+ levels in U87MG and NSP estimated experimentally using NADH/NAD+ measurement kit (Sigma Pvt. Ltd). NADH and NAD+ levels are estimated in pmoles according to the manufacturer's protocol.
